# The Response of *Krascheninnikovia ceratoides* (L.) Gueldenst. to Environmental Changes Since the Mid‐Holocene in the Tibetan Antelope Breeding Ground of the Western Kunlun Mountains

**DOI:** 10.1002/ece3.72900

**Published:** 2026-01-16

**Authors:** Kailing Huang, Fengbing Lai, Mengyu Chen, Ying Song, Shujiang Chen, Zubaydah Wubuaysan, Xiaopeng Zhuang

**Affiliations:** ^1^ Xinjiang Key Laboratory of Arid Zone Lake Environment and Resources Xinjiang Normal University Urumqi China; ^2^ College of Geographical Science and Tourism Xinjiang Normal University Urumqi China; ^3^ Beijing Key Laboratory of Precision Forestry, Forestry College Beijing Forestry University Beijing China

**Keywords:** biomod2, habitat quality, *K. ceratoides*, Western Kunlun Tibetan antelope breeding grounds, XGBoost‐SHAP

## Abstract

*Krascheninnikovia ceratoides* (L.) Gueldenst., the primary food source for the western population of *Pantholops hodgsonii* (Tibetan antelope) during their breeding period in the Western Kunlun Mountains, plays a crucial role in maintaining alpine ecosystems. Herein, we focused on this species and used optimized MaxEnt and biomod2 models to predict its potential distribution ranges during the Mid‐Holocene, current period, and future climate scenarios (RCP2.6, RCP4.5, RCP6.0, RCP8.5), incorporating both climatic and soil factors and performing cross‐validation with the XGBoost‐SHAP model. Additionally, we analyzed the centroid migration characteristics of suitable habitats under different scenarios and identified conservation gaps for Tibetan antelopes using the InVEST Habitat Quality module. The results showed that both biomod2 and XGBoost‐SHAP models exhibited high accuracy: biomod2 had AUC values > 0.98, TSS values > 0.85, and Kappa values > 0.6; the XGBoost‐SHAP model had small RMSE and MAE for both training and test sets, with *R* ^2^ > 0.98. Analysis of environmental factor contributions revealed that mean diurnal range (bio2) had the highest contribution to the distribution of *K. ceratoides* , followed by annual mean temperature (bio1), annual precipitation (bio12), and topsoil clay fraction (t_clay). Currently, the total potential suitable habitat area of this species is 0.5745 × 10^4^ km^2^, accounting for 35.6855% of the study area, and is mainly concentrated in the western and central regions. Among different climate scenarios, the Mid‐Holocene had the largest suitable habitat area (0.7436 × 10^4^ km^2^, 46.1838% of the study area), followed by the RCP4.5 scenario. Warming to some extent promoted the expansion of suitable habitats, but the newly added areas were mostly low suitable habitats, which may face resource competition pressure. With rising temperatures, the centroid of suitable habitats generally migrated from the southeast to high altitude areas in the northwest, then shifted back to the southeast over time. Furthermore, the average habitat quality index of the study area was only 0.277, indicating a relatively harsh ecological environment, and conservation gaps for Tibetan antelopes were concentrated in the southern areas adjacent to existing protected zones. This study provides a scientific basis for the targeted conservation of *K. ceratoides* and the improvement of conservation gaps for Tibetan antelopes.

## Graphical abstract

We analyzed changes in the suitable habitat of *K.ceratoides*, which is the main food source of Tibetan antelopes, within the Tibetan antelope breeding grounds in the Western Kunlun Mountains. The study covered the period from the Mid‐Holocene to future projections and also examined the driving factors and shifting directions of the suitable habitat. Finally, we used in INVEST habitat quality to supplement protection gaps.

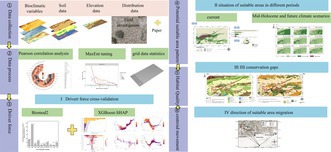

## 1 Introduction

The Mid‐Holocene (6 ka BP) is the most recent warm period. An analysis found through pollen fossils that arid regions in China were dominated by grasslands and forest‐steppes during the Mid‐Holocene (MH), with higher moisture levels than in the Early and Late Holocene (An et al. 2006). Collins showed that vegetation in southern Europe was denser during the MH than today, dominated by temperate vegetation, with widespread sclerophyllous vegetation in the Mediterranean. The distribution and abundance of *Cedrus*, *Abies*, *deciduous* and *evergreen Quercus* were significant in this region (Collins et al. 2012). Hély reconstructed the paleoenvironment of Africa and found that the Sahara Desert was extensively covered with grasslands, shrubs, trees, and wetlands during the MH (Hély and Lézine 2014). According to the Intergovernmental Panel on Climate Change (IPCC) Sixth Assessment Report, the global average surface temperature has increased by 1.1°C over the past more than 100 years (from 1850–1900 to 2011–2020), and is projected to rise by an additional 1°C to 5.7°C by 2081–2100 (depending on greenhouse gas emissions). As global temperatures increase, they will significantly impact the functioning and structure of ecosystems, alter the habitat ranges of many organisms, threaten biodiversity, and even increase the risk of species extinction (Priti et al. 2016).

The view that climate is a key factor affecting species distribution is widely recognized by society (He et al. 2019). Due to limited migration ability, plants are more sensitive to climate change (Tekin et al. 2022). Climate change, involving changes in temperature, precipitation, and atmospheric composition, poses a continuous challenge to plant development and adaptation (Gray and Brady 2016). Plants exhibit dynamic response mechanisms to climate change; increased temperatures lead to a relative enhancement in the abundance of heat‐tolerant plants, while precipitation changes alter the abundance of water‐dependent plants, thereby changing plant community composition (Anderson and Song 2020), meanwhile, functional traits such as leaf morphology, photosynthetic efficiency, and reproductive strategies are affected (Feeley et al. 2020). The geographical range of plants is also undergoing profound shifts. It is projected that plant distributions will migrate to higher altitudes and latitudes, resulting in more climate‐resilient plants expanding their ranges and displacing native plants, reducing their suitable habitats and increasing extinction risks (Garza et al. 2020). Therefore, prospective prediction of plant distributions under climate change can enhance our understanding of biodiversity and ecosystems, which has important implications for the conservation and management of natural resources (He et al. 2023).

As technology advances, species distribution models (SDMs) have been employed to study the geographical distribution of vegetation. Common models include Support Vector Machines, Generalized Linear Models, Maximum Entropy Models, Random Forests, Boosted Regression Trees, and Artificial Neural Networks (Lee‐Yaw et al. 2022; Wang, Yang, et al. 2024). As an important tool in ecological and biogeographical research, species distribution models are based on niche theory. They primarily use specific algorithms to analyze known plant geographical distribution data and corresponding environmental factors, thereby predicting the range of their suitable habitats (Gan et al. 2025). The models are applicable not only to basic ecological research but also to applied research, including identifying key factors influencing species distribution, assessing niche conservatism, determining biological invasion risks, testing biogeographical hypotheses, and predicting species distribution and diversity under global environmental change. Additionally, they are used to study species characteristics such as functional traits, secondary taxa, and genes. When intersecting with other disciplines, they can also provide support for research in population genetics, phylogenetics, phylogeography, and related fields (Guo et al. 2020). The selection of models and parameter settings in species distribution models has a significant impact on modeling results. Therefore, it is crucial to select appropriate statistical algorithms and optimize parameter settings based on the ecophysiological characteristics of the modeling subject (Thibaud et al. 2014). The accuracy of modeling results using the aforementioned common single models still needs improvement. In recent years, an increasing number of species distribution studies have utilized ensemble models, namely the Biomod2 ensemble model, which is more stable and accurate compared to other single models (Jiang et al. 2025). Currently, the Biomod2 platform in R provides Generalized Linear Models, Gradient Boosting Models, Classification and Regression Tree Analysis, Artificial Neural Networks, Surface Distance Envelope, Flexible Discriminant Analysis, Random Forests, Multivariate Adaptive Regression Splines, and Maximum Entropy Algorithms. To date, Biomod2 has been widely used in research on ecological niche modeling and species distribution modeling (Yang, Jia, and Hua 2025).

As a large mammal endemic to the Qinghai‐Tibet Plateau, the Tibetan antelope ( *Pantholops hodgsonii* ) is primarily active in the northern part of the plateau. It typically inhabits flat terrain at elevations of 4000–5000 m with low vegetation coverage, having adapted to the harsh plateau environment characterized by low temperatures, hypoxia, and high ultraviolet radiation. This adaptation is attributed to specialized traits such as enlarged nasal air sacs, dense fur, and robust oxygen transport capacity (Wang, Li, and Tseng 2023). As a national first‐class protected animal in China, Tibetan antelopes have a long population history; fossil evidence indicates their presence on the Qinghai‐Tibet Plateau since at least the Early Pleistocene. Their main activity range is divided into wintering and breeding areas. Female Tibetan antelopes generally migrate to breeding areas in late May to early June and return to wintering areas in early August. Seasonal migration behavior, as a crucial ecological characteristic of the species, is essential for successful reproduction (Ruan et al. 2005). The breeding ground of Tibetan antelopes in the Western Kunlun Mountains serves as the primary breeding area for the western population of Tibetan antelopes, with the breeding peak occurring from late June to early July each year. Approximately 4000–4500 female Tibetan antelopes breed here, whose main food source is *K. ceratoides* (L.) Gueldenst., accounting for about 57%, along with a small amount of gramineous plants (Schaller et al. 2006). *K. ceratoides* is a cushion‐shaped dwarf semi‐shrub belonging to the Krascheninnikovia in the Amaranthaceae, typically reaching a height of 10–25 cm with dense, short branches; annual branches measure 1.5–5 cm in length. It grows on slopes or gravelly flat areas at elevations of 3500–5000 m, and its distribution is concentrated in Gansu (Qilian Mountains), Xinjiang, Qinghai, Tibet, and Nei Mongol (Zhao et al. 2017). *K. ceratoides* is a young plant type emerged from paleo‐Mediterranean elements influenced by the Quaternary glaciation and the uplift of the Qinghai‐Tibet Plateau, and it is an alpine desert species formed by the cold and arid continental climate (Zhuo et al. 2010). It serves as a crucial indicator for studying the formation and evolutionary history of regional floras. Existing studies have mostly focused on *K. ceratoides* , including biogeographical history (Seidl et al. 2020), phylogeny and genetic structure (Qiu 1997), etc. Research on *K. ceratoides* has mostly been mentioned in alpine plant communities (Dong et al. 2024; Jin et al. 2023). However, the distribution pattern of *K. ceratoides* under future climate conditions remains unclear. Therefore, in‐depth exploration of the distribution pattern and migration direction of *K. ceratoides* under different scenarios is of great significance for protecting Tibetan antelopes and maintaining alpine ecosystems. In view of this, we used the biomod2 model to predict the suitable habitat patterns of *K. ceratoides* in the Mid‐Holocene, current, and future climate conditions, and used the XGBoost‐SHAP model to cross‐validate the suitability driving factors. Meanwhile, the InVEST Habitat Quality module was used to improve conservation gaps, and finally, centroid migration analysis was used to determine the centroid migration direction of suitable habitats of *K. ceratoides* in various periods. This research aims to establish a robust foundation for the conservation, management, and habitat evolution studies of *K. ceratoides* .

## 2 Study Area and Materials

### 2.1 Study Area

**FIGURE 1:**
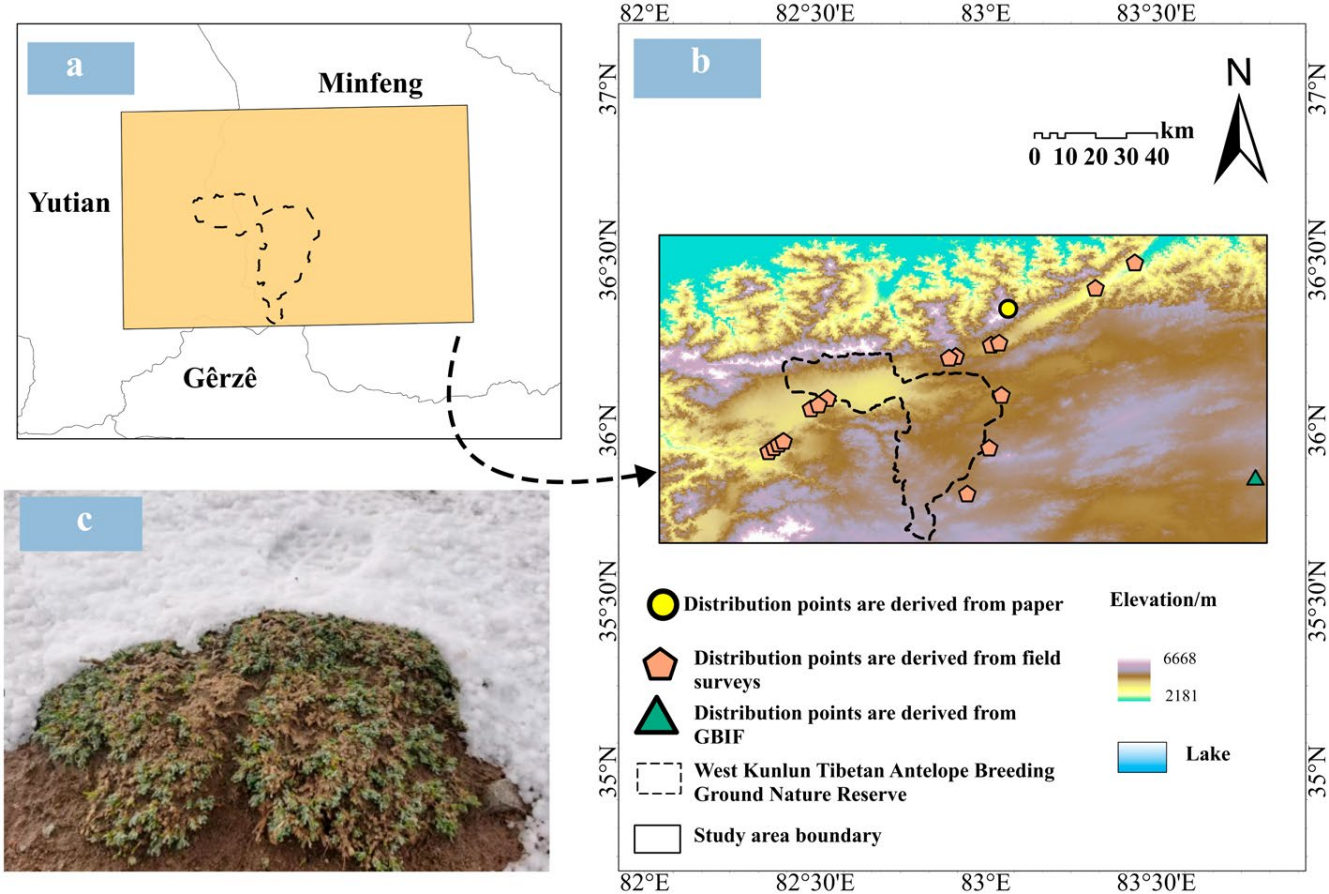
The location of the study area. (a) Location of the study area. (b) Study area. (c) Field photo of *K. ceratoides* by the research team (taken on July 29, 2023). This map is based on a map [GS (2020) 4632] downloaded from the National Geographic Information Public Service Platform website. The base map has not been modified.

The study area includes the Prefecture‐level Nature Reserve for Tibetan Antelope Breeding Grounds in the Western Kunlun Mountains and its surrounding regions, collectively referred to as the Tibetan Antelope Breeding Grounds of the Western Kunlun Mountains. Located on the Qinghai‐Tibet Plateau with an average elevation of 4817 m, this area lies at the junction of three counties: Minfeng County (Xinjiang), Yutian County (Xinjiang), and Gêrzê County (Tibet). It spans approximately 35°39′51.11′′–36°34′21.11′′ N and 82°02′30.28′′–83°49′30.28′′ E, with a perimeter of 521.155 km and a total area of 1.61 × 10^4^ km^2^ (Figure 1). The region has an annual average temperature of −16°C to 8°C, with hottest season temperatures ranging from −5°C to 20°C and coldest season temperatures from −27°C to −5°C. Annual precipitation totals 29–123 mm, with 20–94 mm in the hottest season and 0–2 mm in the coldest season. Characterized by extreme cold and aridity, it exhibits an extremely arid desert climate. Vegetation in this area shows strong cold and drought tolerance, including species such as *Saussurea japonica* (Thunb.) DC., *Cremanthodium reniforme* (DC.) Benth., *Androsace tapete* Maxim., *Stipa roborowskyi* Roshev., and *K. ceratoides* (Jin et al. 2022). Cushion plants dominate the flora, particularly *K. ceratoides* . Soils are primarily sandy with low organic matter content, predominantly classified as alpine desert soil, a calcisols, with partial distributions of saline‐alkaline soil.

### 2.2 Sample Data on the Distribution of *K. ceratoides*

The geographical distribution data of *K. ceratoides* were obtained through the Global Biodiversity Information Facility (GBIF, http://www.gbif.org), two field surveys conducted in July 2023 and June 2025, and a review of literature (Zhao et al. 2017). To ensure the accuracy of the obtained sample point data and avoid errors caused by geographical aggregation, all sample points were subjected to a point thinning test using ENMTools software, with only one distribution point retained per 1 km × 1 km grid (The detailed information of the distribution sites is shown in Table S1).

### 2.3 Data Acquisition Processing

**FIGURE 2:**
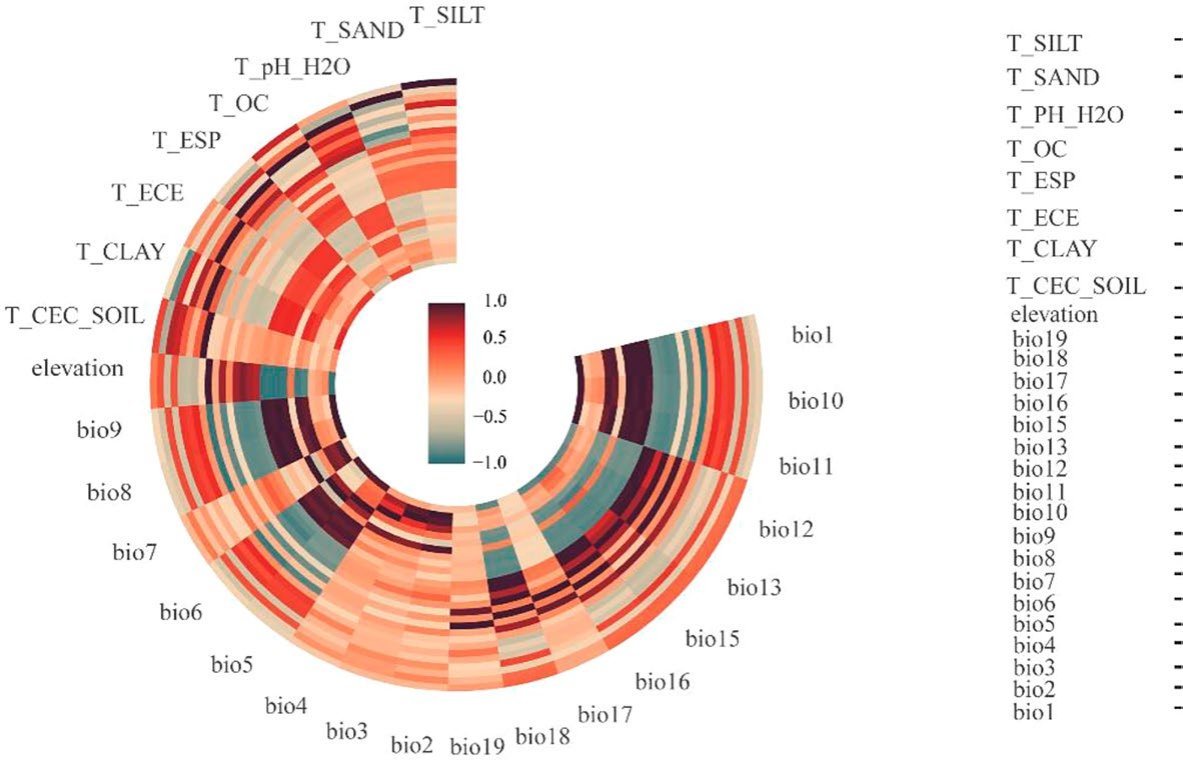
Pearson correlation analysis.

This study included 18 bioclimatic variables, 1 elevation variable, and 8 soil variables. Among these, the bioclimatic factor bio14 (precipitation of the driest month) was excluded because the precipitation in the driest month across the study area was consistently 0 mm, which lacked statistical variability. The bioclimatic data were obtained from the WorldClim database (http://www.worldclim.org) with a resolution of 30′ (approximately 1 km spatial resolution) (Fick and Hijmans 2017). The elevation data were obtained from Geospatial Data Cloud (https://www.gscloud.cn/search). Soil data were derived from the Harmonized World Soil Database v1.2 (https://www.fao.org/soils‐portal/en/), with topsoil at a depth of 0–30 cm selected. The vector boundary data of the prefecture‐level reserve for Tibetan antelope ( *Pantholops hodgsonii* ) breeding grounds in the western Kunlun Mountains and the road network data were obtained from OpenStreetMap (https://www.openstreetmap.org/#map=7/37.262/86.353). The grazing intensity data for the year 2023 were obtained from the public dataset published by Wang, Peng, et al. (2024), where grazing intensity is defined as the cumulative pressure exerted on grassland resources by large herbivores, including cattle, goats, and sheep. Land use data for the year 2023 were acquired from the Resource and Environmental Science Data Platform (https://www.resdc.cn/). The current climate is based on version 2.0 data from 1970 to 2000. The MH and future climate data were generated using the Community Climate System Model version 4 (CCSM4). The Community Climate System Model (CCSM) is a coupled climate model that simulates the Earth's climate system, developed by the National Center for Atmospheric Research (NCAR). The CCSM4 experiments include combinations of human and natural forcings during the 20th century. Anthropogenic forcings include time‐evolving greenhouse gases, tropospheric ozone, stratospheric ozone, and sulfates (Zhao et al. 2021). The random sampling points were created using ArcGIS 10.8, with a point spacing greater than 1 km, resulting in a total of 200 sampling points. These points were combined with environmental factors using the “Extract Multi Values to Points” tool. Subsequent analyses were conducted in R 4.4.1, including Pearson correlation analysis and multicollinearity Variance Inflation Factor (VIF) analysis. Environmental factors with a correlation coefficient |*r*| < 0.8 and VIF < 10 were reserved (Figure 2), where a VIF < 10 indicates no multicollinearity among factors. The bio1 and bio12 serve as comprehensive indicators of global climate change, predominantly influencing species' optimal distribution at broad spatial scales (Liu et al. 2020). The distribution area of *K. ceratoides* is located on the Qinghai‐Tibet Plateau. Plants endemic to this region typically exhibit a narrow tolerance range to temperature fluctuations and are consequently more susceptible to influences from bio2 and bio4. Precipitation in this region is concentrated between June and August. However, the vegetation germination period occurs in March, which is characterized by a relatively arid environmental state. During this critical phase, soil water content and temperature are crucial for vegetation development. Based on the above information, and considering the specific biological characteristics of *K. ceratoides* and its soil environment (Wu et al. 2024; Liu 2004; Xia et al. 2024). Ultimately, 10 environmental variables were retained, consisting of 5 bioclimatic variables and 5 soil variables (Table 1). All raster data were unified to a resolution of 30′ with the projection coordinate system WGS_1984_UTM_Zone_45N.

**TABLE 1:** Environmental variables used for model construction.

Environmental variables	Code	Unit
bio1	Annual mean temperature	°C
bio2	Mean diurnal range (Mean of monthly [max temp–min temp])	°C
bio4	Temperature seasonality (standard deviation × 100)	°C
bio12	Annual precipitation	mm
bio17	Precipitation of driest quarter	mm
T_CEC_SOIL	Topsoil cation exchange capacity	cmol/kg
T_CLAY	Topsoil clay fraction	%weight
T_ECE	Topsoil salinity (Elco)	dS/m
T_ESP	Topsoil sodicity (ESP)	%
T_SILT	Topsoil silt fraction	°C

### 2.4 MaxEnt Model Construction and Parameter Optimization

**FIGURE 3:**
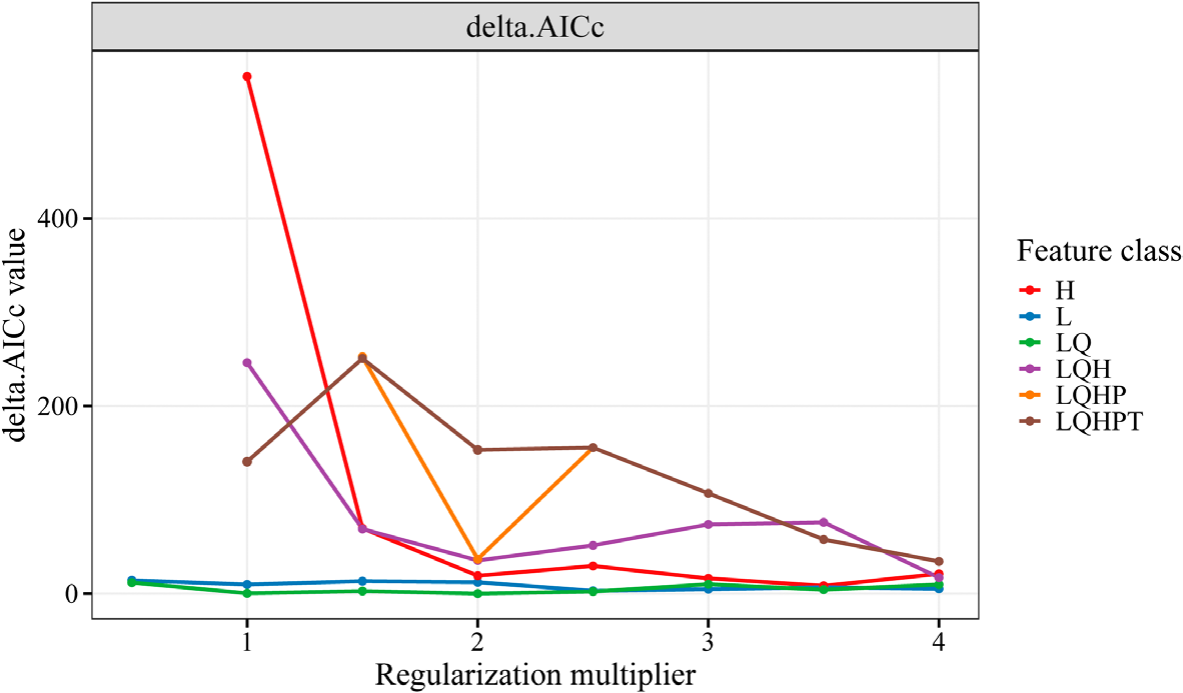
MaxEnt tuning selection results.

To reduce overfitting and improve predictive performance, the default parameters in Maxent have been carefully calibrated based on numerous empirical tuning studies (Li and Zhou 2025). In R 4.4.1, the ENMeval package was used for optimal parameter selection, modifying and adjusting two parameters: Feature Combination (FC) and Regularization Multiplier (RM). The MaxEnt model provides five feature types: Linear features (L), Quadratic features (Q), Product features (P), Threshold features (T), and Hinge features (H). This study tested six feature combinations: H, L, LQ, LQH, LQHP, and LQHPT. The regularization multiplier (RM) was set from 0.5 to 4 with increments of 0.5, resulting in eight regularization levels, totaling 48 parameter combinations. Additionally, adjustments were made to random background points, and after multiple comparisons, the training receiver operating characteristic curve (AUC) was highest when 700 random background points were used. The performance evaluation of the model in this study was primarily based on several key metrics: the difference between training AUC and test AUC (AUC.diff.Avg), the mean 10% test omission rate (Mean.or10), and the corrected Akaike Information Criterion (delta.AICc). Among these, AUC.diff.Avg and Mean.or10 were used to assess the model's fitting degree to species distribution points, with smaller values indicating a higher fitting degree. delta.AICc was used to evaluate the model's complexity and transferability; generally, the model is considered optimal when delta.AICc was minimized (delta.AICc = 0) (Figure 3) (Yang, Ding, and Tian 2025). Ultimately, the parameter combination model with FC = LQ, RM = 2, and 700 background points were selected.

### 2.5 Construction of the biomod2 Model

**FIGURE 4:**
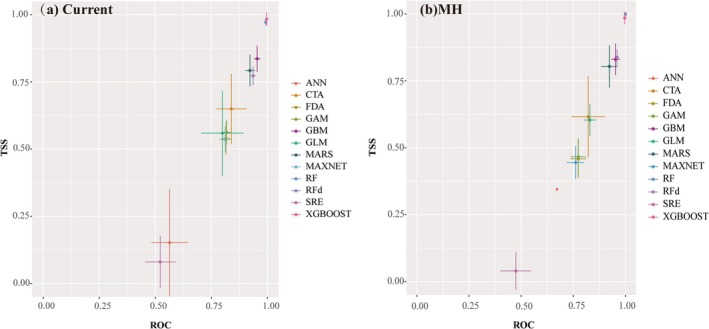
Single model accuracy. (a) Current. (b) MH.

Species distribution modeling was performed using the biomod2 package in R4.4.1. The presence records were partitioned into training (75%) and testing (25%) datasets. To ensure robustness, the partitioning procedure was randomly repeated three times. For each partitioning replicate, the models were run 10 times with random initializations. Additionally, 700 pseudo‐absence points were randomly generated for model calibration. We employed an ensemble of 12 distinct modeling algorithms: MaxEnt (Maximum Entropy, with tuned parameters), Classification Tree Analysis (CTA), Flexible Discriminant Analysis (FDA), Generalized Additive Model (GAM), Generalized Boosted Model (GBM), Generalized Linear Model (GLM), Multivariate Adaptive Regression Splines (MARS), Random Forest (RF), Random Forest with a sampling approach (RFD), Surface Range Envelope (SRE), Extreme Gradient Boosting (XGBOOST), and Artificial Neural Network (ANN). All models were run with default software settings, except for MaxEnt (Maximum Entropy), which employed tuned parameters, model calibration and evaluation were conducted through fivefold cross‐validation. The true skill statistic (TSS) and the area under the AUC were used to evaluate model accuracy, with the Kappa coefficient additionally incorporated for accuracy validation during the final model selection. The AUC value ranges from 0 to 1, with higher values indicating greater accuracy of model predictions. Typically, an AUC value between 0.5 and 0.6 indicates a failed prediction; 0.6–0.7 indicates poor performance; 0.7–0.8 indicates moderate performance; 0.8–0.9 indicates good performance; and an AUC > 0.9 indicates excellent performance (Qiao et al. 2025). The TSS accounts for the mean absence error and is unaffected by the size of the validation dataset. Models can be categorized into five groups based on TSS: TSS > 0.8 for excellent models, 0.6–0.8 for good models, 0.4–0.6 for moderate models, 0.2–0.4 for poor models, and TSS < 0.2 for failed models (Lasram et al. 2010). The Kappa coefficient ranges from −1 to 1, where 1 indicates 100% agreement between predictions and reality, and −1 indicates 100% disagreement (Huang et al. 2023). To ensure robustness and accuracy, we selected optimal single models that met the dual thresholds of TSS > 0.7 and AUC > 0.8, based on criteria established in prior studies (Lan and Zhang 2025; Zhao et al. 2025), to serve as the base models (Figure 4). The ensemble modeling algorithm was used to simulate the potential suitable distribution areas of *K. ceratoides* in the Tibetan antelope breeding grounds of the Western Kunlun Mountains under MH, current, and future climate scenarios. Finally, centroid migration analysis of suitable habitats was performed on the results using ArcGIS 10.8.

### 2.6 Habitat Quality

To better identify the overall ecological environment and protection gaps in the study area, we established a 20 km buffer zone for analysis using ArcGIS 10.8, and evaluated the habitat quality of the Tibetan antelope breeding grounds in the Western Kunlun Mountains through the InVEST model (version 3.14.2). The Habitat Quality module primarily utilizes quantitative ecological parameters and land use models to assess habitat degradation and habitat quality indices for each land use type based on biodiversity threat information (Wang, Hao, et al. 2024). One advantage of this method is its ability to replace detailed field surveys and quantify changes in habitat quality within a short period (Xiao et al. 2023). Based on the InVEST reference manual and previous studies (Jiang et al. 2024; Liu et al. 2021), we determined the parameters for the InVEST model's Habitat Quality module (as shown in Tables S2 and S3), and extracted six threat sources (Bare land, Dry land, Grazing, Road, Rural settlements, Saline land) to evaluate their impacts on habitat quality. The habitat quality index was calculated using the following formula (Guo et al. 2023).

(1) $$Q_{\mathrm{xj}}=H_{j}\left(1-\frac{D_{\mathrm{xj}}^{z}}{D_{\mathrm{xj}}^{z}+k^{z}}\right)$$

In the formula, $Q_{\mathrm{xj}}$ represents the habitat quality of land use type j in grid cell x, ranging from 0 to 1, with higher values indicating better habitat quality, $H_{j}$ denotes the habitat suitability of land use type j, z is the normalization exponent, set to 2.5, k is the half‐saturation constant, set to 0.05, and $D_{\mathrm{xj}}$ represents the degree of stress experienced by grid cell x in land use type j.

### 2.7 Factors Influencing the Divergence of Potential Suitability Zones

XGBoost, as a tree‐based machine learning model, constructs multiple estimators through residual fitting. Each iteration generates an estimator trained on the basis of the previous round, enabling the model's predictions to approximate actual values to the greatest extent. Compared with traditional tree‐based ensemble learning models such as Random Forest (RF) and Gradient Boosting Decision Tree (GBDT), XGBoost exhibits significant robustness and flexibility in handling missing values and large‐scale datasets. Despite its advantages, including high performance in speed and accuracy, scalability, ability to manage numerical and categorical data, and resistance to overfitting (Wang, Li, Yuan, et al. 2023), XGBoost has low interpretability and is often regarded as a black‐box model. The feature importance values of the XGBoost model can only rank environmental variables by importance but cannot explain the relationships between individual features and prediction results. Therefore, SHAP is generally introduced to interpret model outcomes (Liu et al. 2024). Based on the Shapley value principle in game theory, SHAP effectively explains the prediction process and feature importance of machine learning models. SHAP values quantify the contribution of individual input features to model predictions, explain the thresholds and interaction effects between explanatory variables and the explained variable (Wang, Li, et al. 2025). In the construction of the XGBoost model, adjusting hyperparameters is crucial for suppressing model overfitting. We optimized the hyperparameter configuration of the XGBoost model through grid search and 10‐fold cross‐validation (Syarif et al. 2016), by setting ranges and step sizes for key parameters to select the optimal ones. Ultimately, the best hyperparameter combination for the XGBoost model in this study was determined as: max_depth = 10, eta = 0.1, nrounds = 200, colsample_bytree = 0.8, min_child_weight = 1, subsample = 0.7, gamma = 0.1, with other parameters set to default values.

For evaluating model performance, the coefficient of determination *R* ^2^, mean absolute error (MAE), and root mean squared error (RMSE) are commonly used. The value range of *R* ^2^ is (−∞, 1], the closer the value is to 1, the better the model performance, indicating that the predicted results are closer to the true values. MAE and RMSE measure the difference between predicted and true values; smaller values indicate higher model accuracy (Wang, Zhang, and Li 2025). In this study, we used XGBoost‐SHAP to validate the suitability driving factors predicted by the biomod2 model, conducted zonal statistics on the suitability index and driving factors using a grid (900 × 1000 m) in ArcGIS 10.8, and then performed the analysis in R 4.4.1.

## 3 Results and Analysis

### 3.1 Model Accuracy Test

The MAXENT model was tuned, and after setting the optimized parameter combination of FC = LQ and RM = 2 to run the model, compared with the default parameter model, the tuned model showed lower values of AUC.diff.Avg and Mean.or10 (Table 2), indicating that the tuned model is more accurate in predicting suitable areas. When conducting biomod2 analysis, we screened individual models with AUC > 0.8 and TSS > 0.7, and constructed an ensemble model using five individual models: XGBOOST, RF, RFd, GBM, and MARS. The ensemble model results were constructed using four methods: cumulative averaging (EMca), simple averaging (EMmean), median (EMmedian), and weighted averaging (EMwmean). Since the AUC value is not affected by diagnostic thresholds and is insensitive to species distribution rates, it can comprehensively compare the accuracy of diagnostic tests (Liu et al. 2025). Based on the highest AUC value, with TSS and Kappa values as supplements, the EMcaByROC model was ultimately selected to predict the potential suitable areas of *K. ceratoides*. For predicting current and future suitable areas, the AUC = 0.99, TSS = 0.929, and Kappa = 0.628, for predicting the MH suitable areas, the AUC = 0.982, TSS = 0.875, and Kappa = 0.643. All indicators met good standards, indicating that the prediction results are accurate and reliable.

**TABLE 2:** The comparison between the default and optimized MaxEnt models.

Type	FC	RM	AUC.diff.Avg	Mean.or10	delta.AICc
Default	LQHP	1	0.19	0.278	92.926
Optimized	LQ	2	0.189	0.167	0

### 3.2 Importance of the Environmental Variable

**FIGURE 5:**
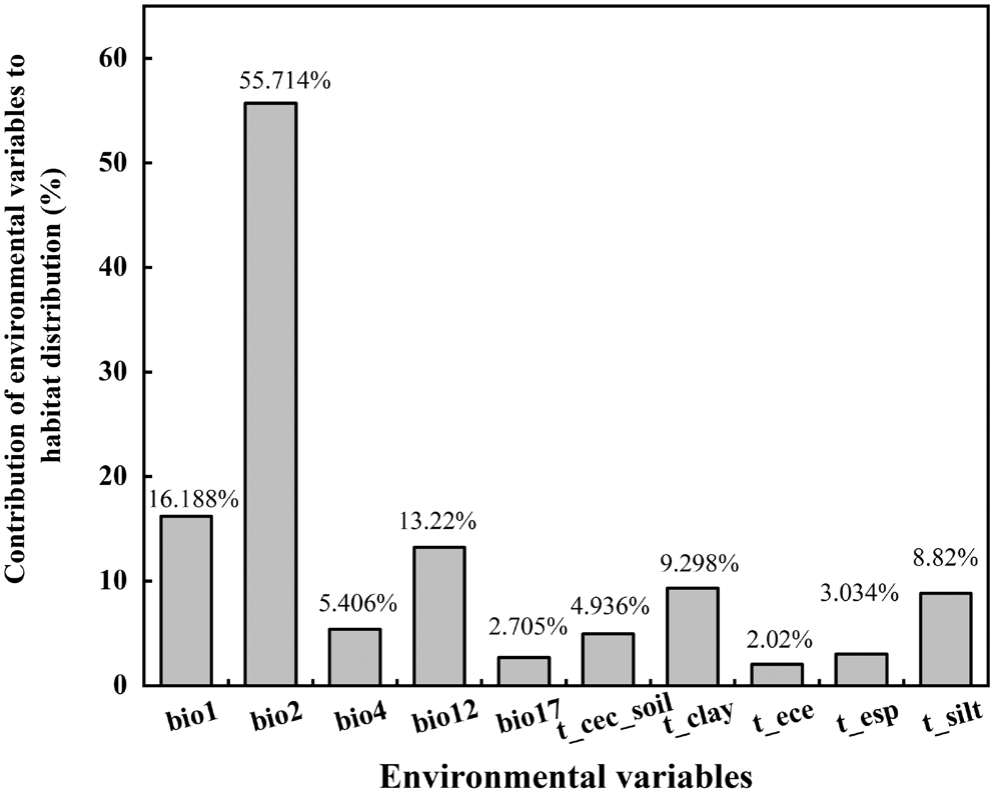
The contribution rates of different environmental variables to the habitat distribution of *K. ceratoides*.

According to the importance assessment of environmental variables derived from the integrated model, the contribution ratios of different environmental factors to the habitat distribution of *K. ceratoides* were illustrated in Figure 5. Key variables influencing *K. ceratoides* distribution were identified based on their contribution ratios, ranked in descending order as: bio2, bio1, bio12, t_clay, t_silt, bio4, t_cec_soil, t_esp, bio17, and t_ece. Specifically, bio2 accounted for 55.7137%, bio1 for 16.1883%, and bio12 for 13.22%, with a cumulative contribution ratio of 85.1219%. These results indicate that temperature and precipitation variables serve as the dominant environmental factors regulating the habitat suitability of *K. ceratoides*.

### 3.3 The Driving Factors of Suitable Habitat of *K. ceratoides* Based on XGBoost‐SHAP

#### 3.3.1 The Accuracy Evaluation of the XGBoost‐SHAP Model

**FIGURE 6:**
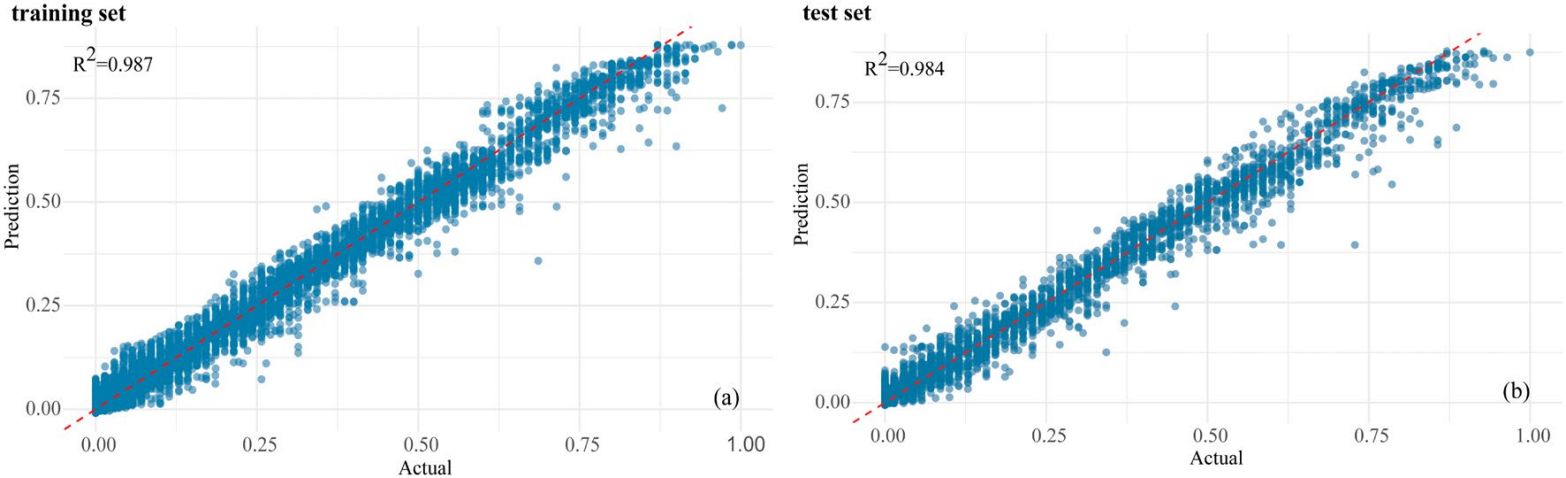
Fitting situation. (a) Training set. (b) Test set.

The XGBoost‐SHAP model was employed to predict the suitability driving factors of *K. ceratoides*, demonstrating favorable predictive performance on both the training and test sets. For the test set, the RMSE was 0.032, the MAE was 0.02, and the *R* ^2^ was 0.984. For the training set, the RMSE was 0.0282, the MAE was 0.0186, and the *R* ^2^ was 0.987 (Figure 6). The RMSE and MAE of the test set were 0.0038 and 0.0014 higher than those of the training set, respectively, with a 0.003 difference in *R* ^2^, indicating slightly better predictive performance of the training set compared to the test set. The low RMSE and MAE values suggest a high degree of consistency between the model's predictions and actual observations, while the *R* ^2^ value approaching one further indicates good model fit to the data, confirming that this model can be used to explain the suitability driving forces of *K. ceratoides* .

#### 3.3.2 The Interpretive Analysis of the XGBoost‐SHAP Model

**FIGURE 7:**
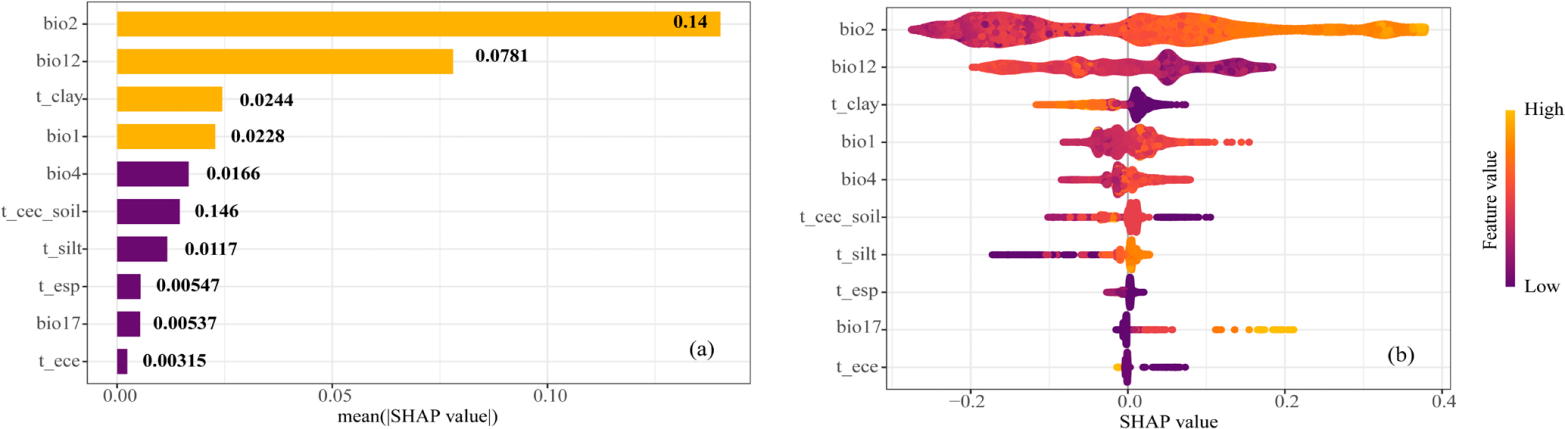
SHAP value visualization. (a) Driving factors importance ranking. (b) Overall sample SHAP value.

**FIGURE 8:**
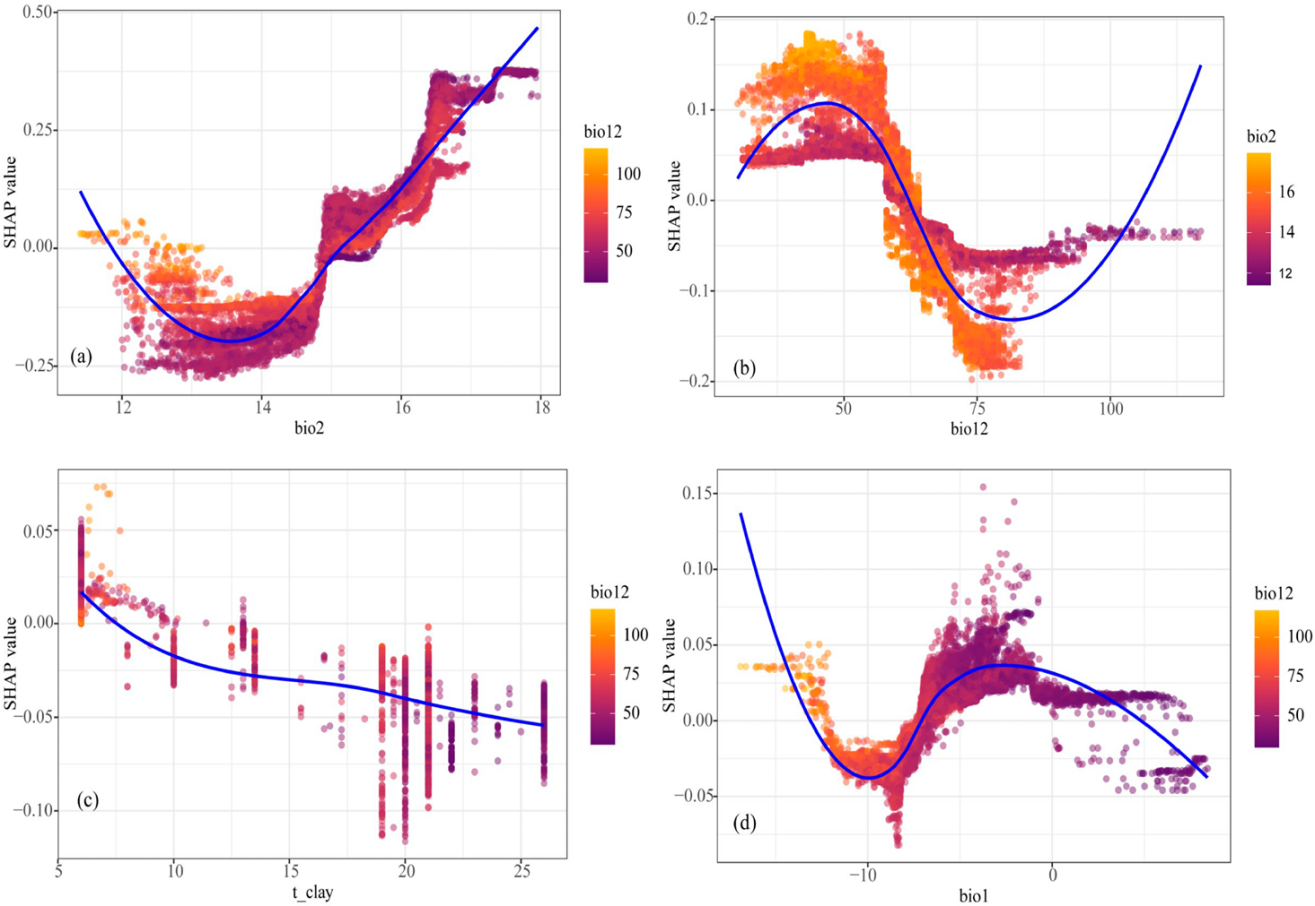
SHAP dependence plot.

The 10 screened environmental factors were subjected to suitability driving force analysis. As shown in Figure 7a, the magnitude of the factor SHAP values (absolute value) indicates their contribution to suitability, with higher values representing a greater contribution. The environmental factor with the highest SHAP value was bio2 (0.14), followed by bio12 (0.0781), t_clay (0.0244), bio1 (0.0228), bio4 (0.0166), t_cec_soil (0.0146), t_silt (0.0117), bio17 (0.00547), t_esp (0.00537), and t_ece (0.00315). Figure 7b further illustrates the impact of each factor on suitability. The *x*‐axis represents the SHAP value, where negative values indicate that the factor characteristic values exert a adverse impact on the results, and positive values indicate a beneficial impact. The larger the absolute SHAP value of a feature, the more it suggests that the feature has a greater influence on the prediction model. Purple indicates low feature values, and yellow indicates high feature values. bio2 and bio17 generally exhibit a positive relationship with suitability, meaning that as the factor characteristic values increase, the SHAP values also increase. In contrast, bio12, t_clay, t_esp, and t_ece show a roughly negative relationship: larger factor characteristic values correspond to lower SHAP values. For bio1 and bio4, SHAP values increase with their characteristic values within a certain range, indicating a positive correlation, but this shifts to a negative correlation beyond that range. The remaining factors (t_cec_soil, t_silt) display complex non‐linear relationships. Based on the ranking of SHAP value importance, we selected the top four dominant factors (bio2, bio12, t_clay, and bio1) to generate SHAP dependence plots (Figure 8), illustrating both the independent effects of individual environmental factors and the combined effects of two closely related factors. A Locally Weighted Scatterplot Smoothing (LOESS) method was used to establish fitted curves, represented by blue lines, to illustrate the trend of individual features' impact on SHAP values, the results indicate that the most critical feature affecting the habitat suitability of *K. ceratoides* is bio2, which exhibits a positive relationship with suitability. As bio2 increases, suitability improves. Additionally, lower bio12 and higher bio2 values enhance suitability probability; however, when bio12 increases, smaller bio2 values still moderately promote suitability. Suitability shows a trend of initial increase, subsequent decrease, and then another increase with bio12. Suitability improves with larger bio2 and reduced bio12, but may also increase when both bio12 and bio2 decrease. t_clay has a significant negative impact on suitability: as t_clay increases, suitability declines. Suitability improves when bio12 decreases and t_clay is low. With increasing bio1, suitability initially decreases, then increases, and subsequently decreases again. Suitability may also improve when bio1 rises to a certain level accompanied by reduced bio12, while lower bio1 combined with increased bio12 can also enhance suitability. These findings suggest that each driving factor has specific critical thresholds for promoting suitability, with temperature and precipitation playing dominant roles.

### 3.4 Spatial Pattern Changes of Suitable Habitats for *K. ceratoides*

#### 3.4.1 Potential Suitable Areas for *K. ceratoides* Under Current Climate Conditions

**FIGURE 9:**
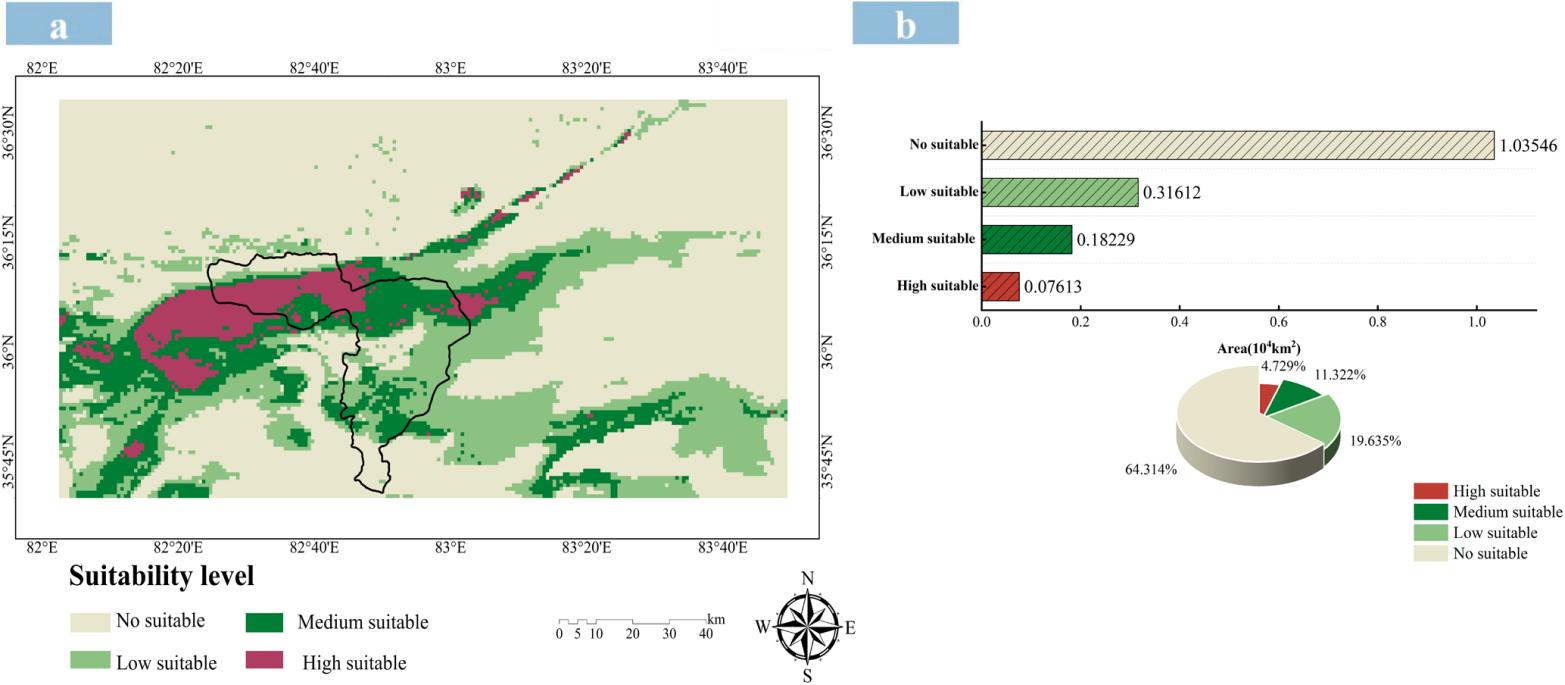
The suitable habitat of the *K. ceratoides* under current climatic conditions. (a) Distribution pattern under current climatic conditions. (b) Area of each suitability grade. (c) The proportion of area in each suitability grade.

**FIGURE 10:**
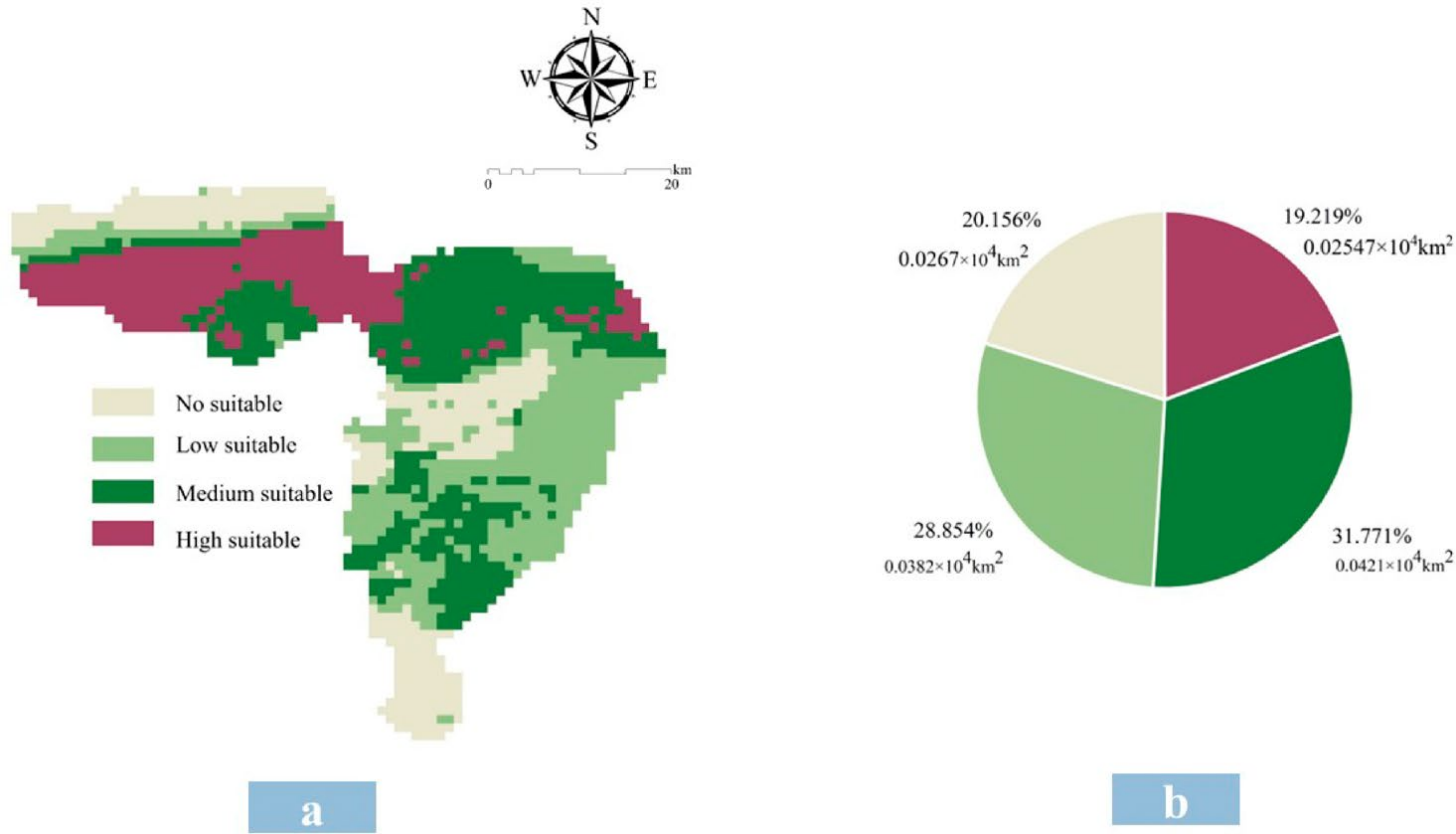
The suitable habitat of *K. ceratoides in* protected areas under current climate conditions. (a) The distribution pattern of suitable habitats in protected areas. (b) The area and proportion of suitability grades.

Reclassify the habitat suitability index P into equal intervals, which are respectively No suitable (≤ 0.25), Low suitable (0.25 < *p* ≤ 0.5), Medium suitable (0.5 < *p* ≤ 0.75), and High suitable (> 0.75). Under the current climate (1970–2000), the total area of suitable habitats for *K. ceratoides* in the study area is 0.5745 × 10^4^ km^2^, accounting for 35.6855%. Among this, the area of high suitability is 0.0761 × 10^4^ km^2^, medium suitability is 0.1823 × 10^4^ km^2^, and low suitability is 0.3161 × 10^4^ km^2^, accounting for 4.723%, 11.322%, and 19.634% of the total study area, respectively. Suitable potential habitat are mostly concentrated in the central and southern regions. The area of unsuitable habitats is 1.0355 × 10^4^ km^2^, mainly distributed in the northern and eastern regions (Figure 9). In the Prefectural‐Level Protected Area for Tibetan Antelope Breeding Grounds in the Western Kunlun Mountains, the total potential suitable habitat area for *K. ceratoides* is 0.1058 × 10^4^ km^2^, accounting for 79.8438% of the total protected area. Among this, the low and medium suitability areas are relatively large, accounting for 28.8541% and 31.7708% of the total protected area, respectively, while the unsuitable area is only 0.0267 × 10^4^ km^2^ (Figure 10). The protected area is relatively suitable for the growth of *K. ceratoides* , which is of great significance for meeting the nutritional needs of Tibetan antelopes after reproduction.

#### 3.4.2 The Potential Suitable Habitat Under MH and Future Climate Conditions

**FIGURE 11:**
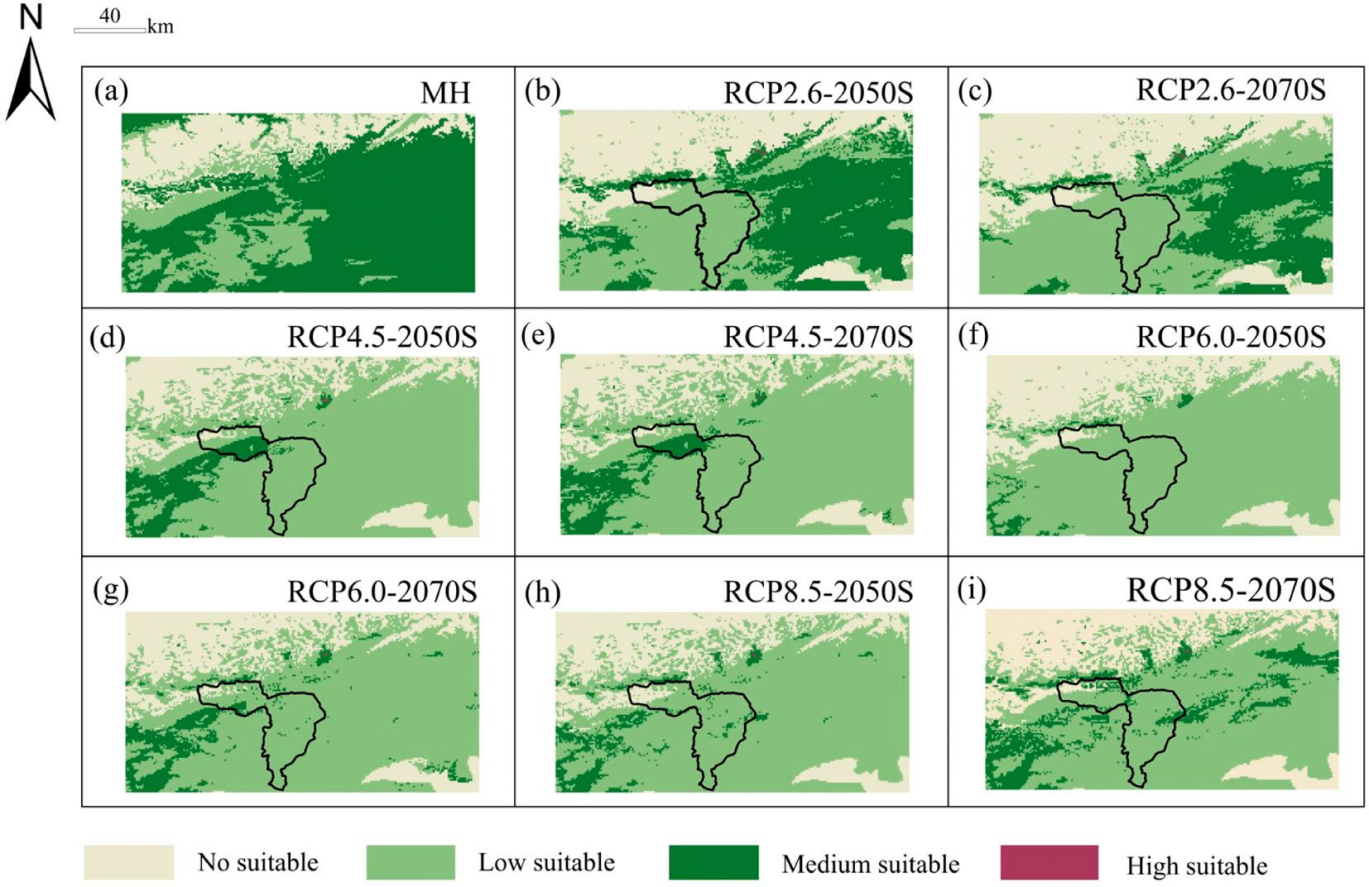
Potential distribution of *K. ceratoides* under MH and future climate conditions.

Select the Mid‐Holocene period (6 ka BP) and the two time periods of 2041–2060 and 2061–2080 under different greenhouse gas emission scenarios (RCP2.6, RCP4.5, RCP6.0, RCP8.5), and use ensemble models to predict the distribution pattern of potential suitable habitats of *K. ceratoides* (Figure 11). During the MH period, the suitable habitat area of *K. ceratoides* was 1.313 × 10^4^ km^2^, dominated by moderately suitable areas, which were mostly concentrated in the eastern part. This region was relatively suitable for the growth of *K. ceratoides* during this period. Under the four future scenarios in different periods, the area of highly suitable habitats will be significantly reduced or even lost. The area of moderately suitable habitats will decrease, with a reduction rate of 29.31%–83% and a reduction area of 0.0534 × 10^4^ km^2^–0.1513 × 10^4^ km^2^ during 2041–2060, and a reduction rate of 28.096%–34.873% and a reduction area of 0.0512 × 10^4^ km^2^–0.0636 × 10^4^ km^2^ during 2061–2080. However, under the RCP2.6 scenario, the moderately suitable habitats increased in both time periods, with increases of 174.8201% and 100.5301% respectively. In addition, under the RCP8.5 scenario, the moderately suitable habitats had a growth rate of 10.3749% during 2061–2080. The area of lowly suitable habitats will expand, with an expansion rate of 8.6977%–258.2096% and an expansion area ranging from 0.05 × 10^4^ km^2^ to 0.8163 × 10^4^ km^2^ during 2041–2060; during 2061–2080, the expansion rate will be 31.8%–258.69% and the expansion area will be 0.1827 × 10^4^ km^2^–0.8178 × 10^4^ km^2^. Unsuitable habitats decreased in both periods (Table 3). In general, with the increase of greenhouse gas concentrations, the areas of unsuitable, highly suitable, and moderately suitable habitats have decreased, while the area of lowly suitable habitats has increased, indicating that rising temperatures exert stress on the growth of *K. ceratoides* .

**TABLE 3:** Area of each suitability zone under Mid‐Holocene and future climate (retaining four decimal places) × 10^4^ km^2^.

	No suitable	Low suitable	Medium suitable	High suitable
MH	0.297	0.3516	0.9615	—
RCP2.6‐2050S	0.484	0.625	0.501	0.0006
RCP2.6‐2070S	0.4865	0.7572	0.3655	0.0007
RCP4.5‐2050S	0.3556	1.1252	0.1289	0.0003
RCP4.5‐2070S	0.3449	1.1339	0.1311	0.0001
RCP6.0‐2050S	0.4466	1.1324	0.031	—
RCP6.0‐2070S	0.3842	1.1068	0.1187	0.0002
RCP8.5‐2050S	0.403	1.1148	0.0919	0.0003
RCP8.5‐2070S	0.4056	1.003	0.2012	0.0002

*Note:* “—” indicates no data.

**FIGURE 12:**
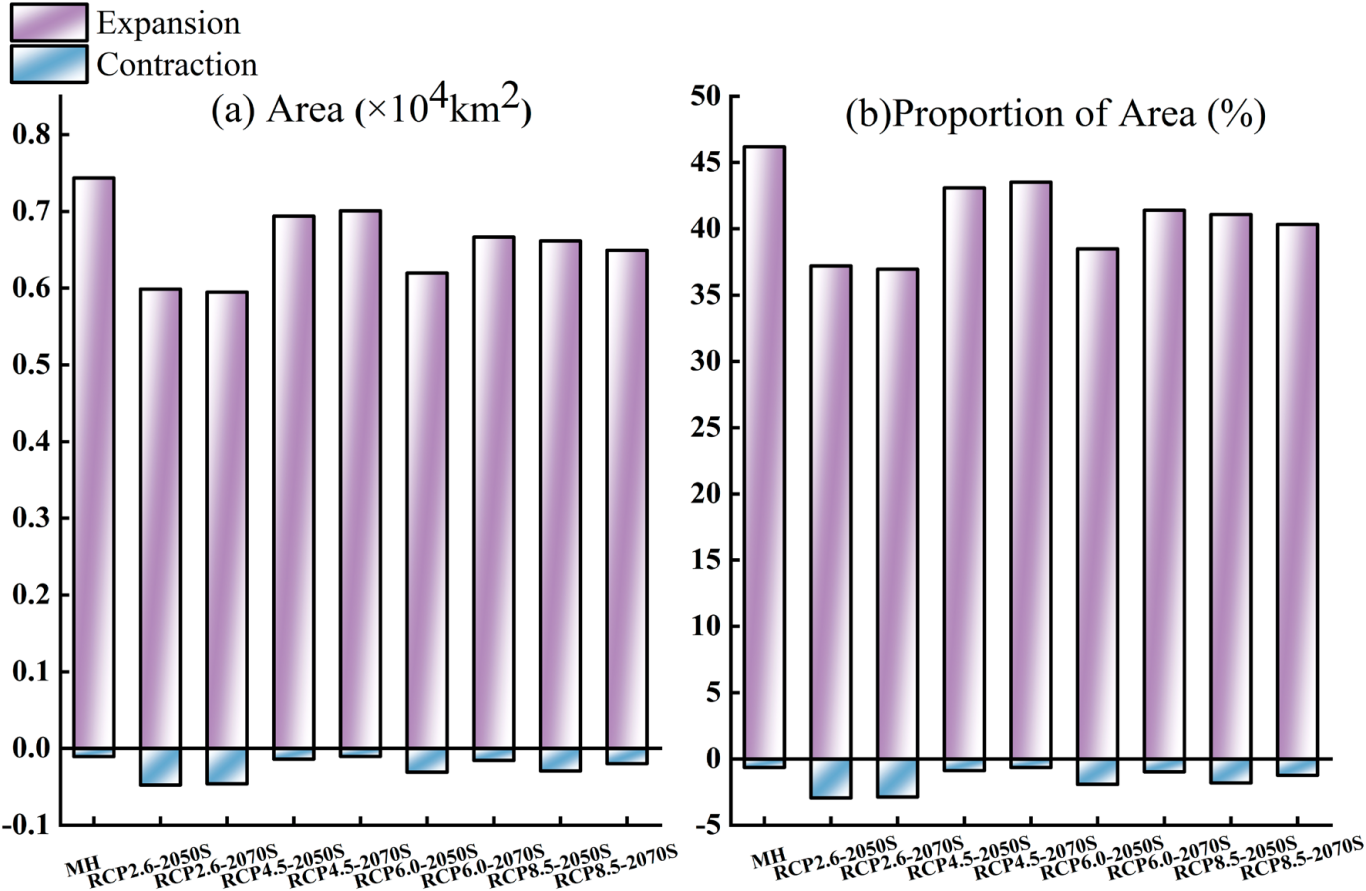
Changes in suitable habitats during the MH and future periods. (a) Area. (b) Proportion of area.

**FIGURE 13:**
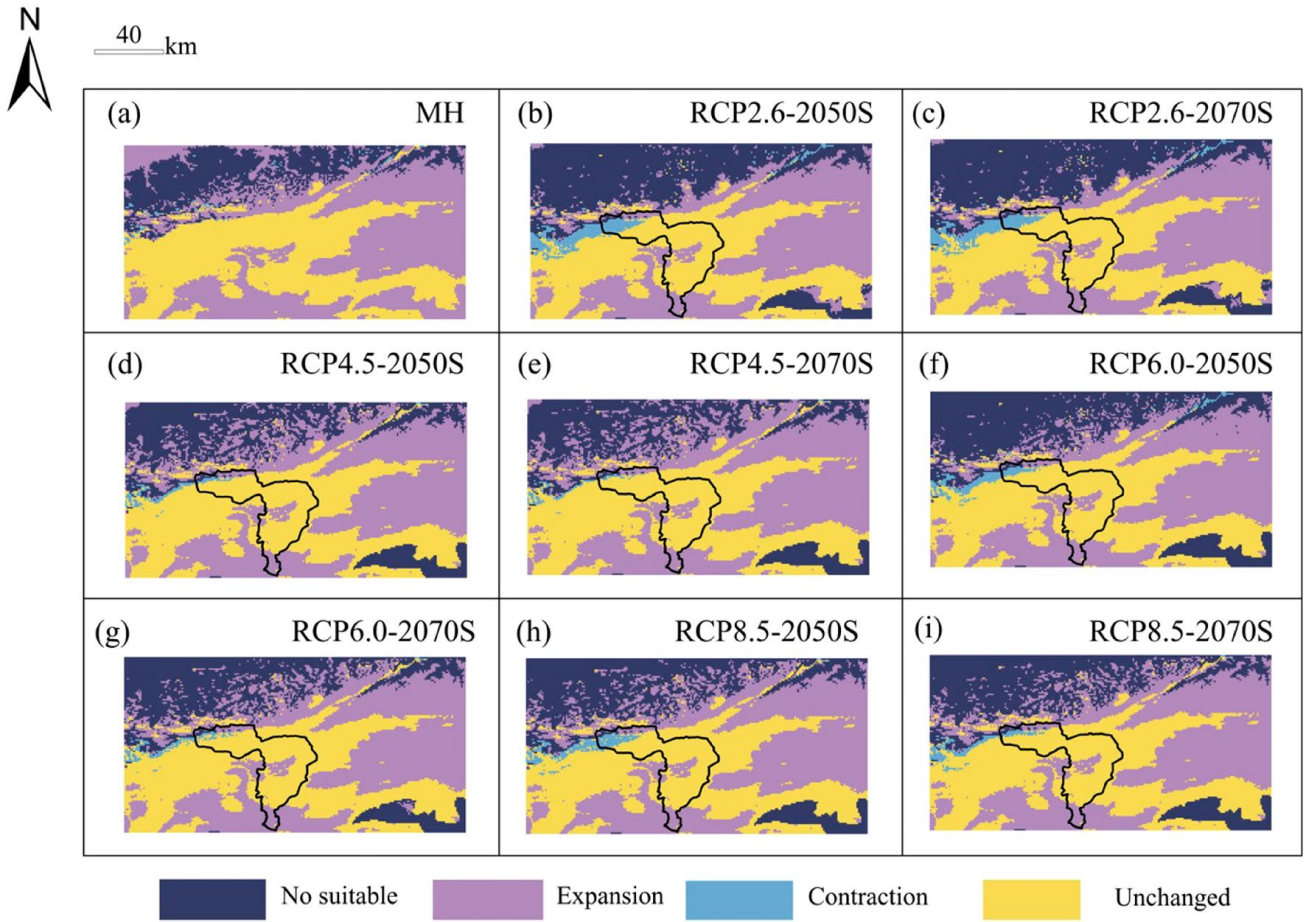
(a–i) Pattern changes of suitable habitats under different scenarios.

Compared with the current climate, the MH period may be more suitable for the growth of *K. ceratoides*, with an increased area of suitable habitats of 0.7436 × 10^4^ km^2^, accounting for 46.1838%. It is projected that the total area of suitable habitats of *K. ceratoides* will expand under four different scenarios. Among which, during the period 2041–2060, the growth rate is the highest under the RCP4.5 scenario, followed by the RCP8.5, RCP6.0, and RCP2.6 scenarios. The expansion areas are 0.6937 × 10^4^, 0.6614 × 10^4^, 0.6195 × 10^4^, and 0.5989 × 10^4^ km^2^ in sequence, with expansion rates of 43.085%, 41.0786%, 38.4807%, and 37.1961% respectively. During the period 2061–2080, the growth rates are in the order of RCP4.5, RCP6.0, RCP8.5, and RCP2.6, with expansion areas of 0.7006 × 10^4^, 0.6666 × 10^4^, 0.6494 × 10^4^, and 0.5949 × 10^4^ km^2^ in sequence, and expansion rates of 43.518%, 41.4044%, 40.3327%, and 36.9491%, respectively (Figure 12). Under the same scenario, the change in the range of suitable habitats between different periods (2041–2060 and 2061–2080) is not significant. However, with the increase in greenhouse gas concentrations, the distribution pattern of suitable habitats changes significantly, especially the expansion of suitable habitats to the northern high elevation areas. This suggests that under the RCP4.5 scenario, environmental conditions such as temperature and precipitation may cause a shift in the ecological niche of *K. ceratoides* (Figure 13). In addition, the suitable habitats of *K. ceratoides* in protected areas show a certain degree of reduction under different scenarios, concentrated in the northern part of the protected areas.

### 3.5 Predicting Centroid Changes of Potential Suitable Areas of *K. ceratoides* Based on Different Climate Scenarios

**FIGURE 14:**
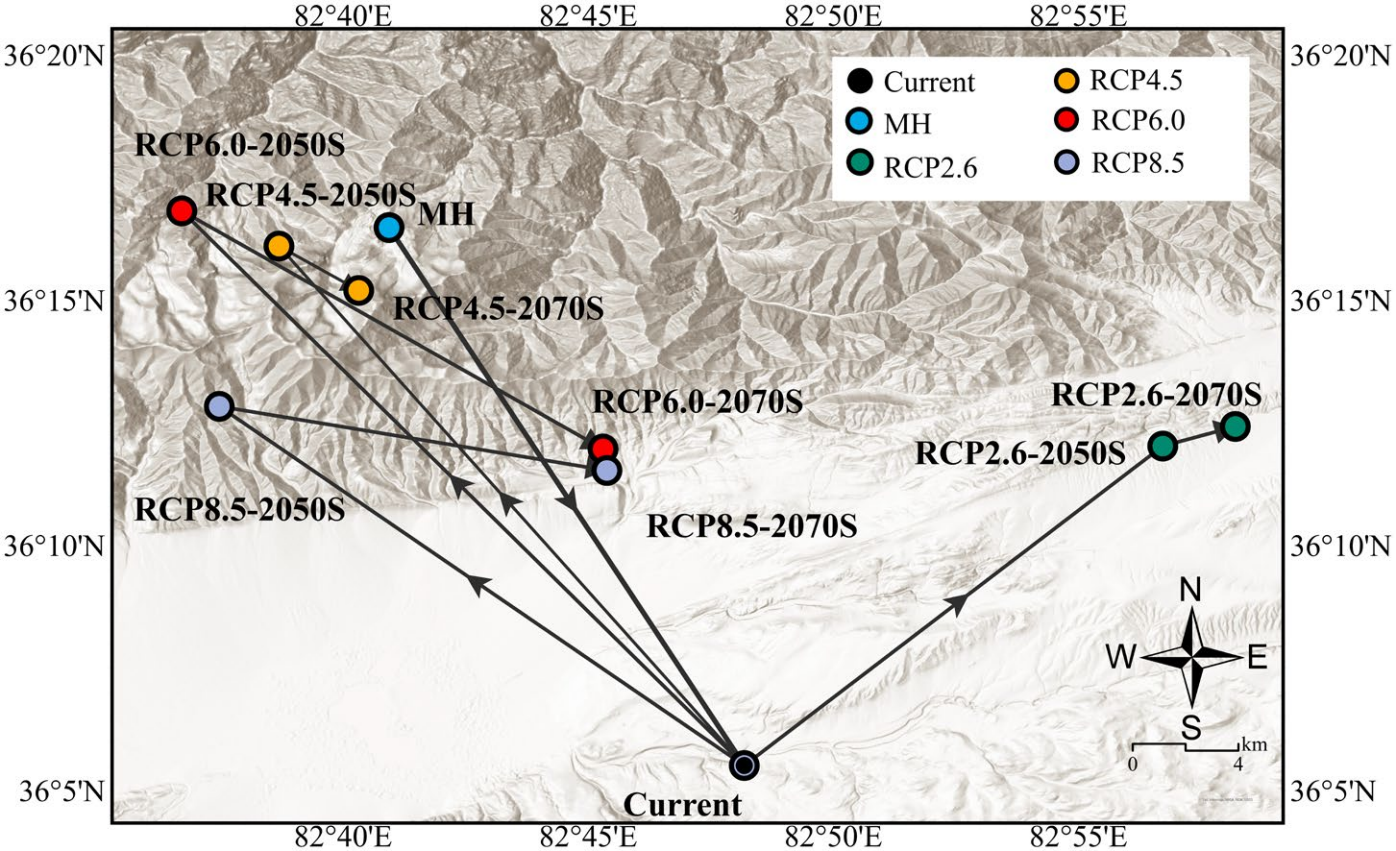
Centroid distribution of suitable habitats for *K. ceratoides* .

We calculated the centroid positions of suitable habitats for *K. ceratoides* under different climate scenarios, along with the direction and distance of centroid movement (Figure 14; Table S4). From the MH to the present, the centroid generally migrated from northwest to southeast by 23.045 km. The current centroid of the suitable area of *K. ceratoides* is located at 82°48′11.106′′ E, 36°5′31.07′′ N. Under different climate scenarios, the centroid migration distance varies. Under the RCP2.6 scenario, *K. ceratoides* first migrated northeastward to 82°56′43.267′′ E, 36°12′1.868′′ N with a migration distance of 17.602 km, and then eastward to 82°58′12.151′′ E, 36°12′25.816′′ N with a migration distance of 2.343 km. Under the RCP4.5 scenario, it first migrated northwestward to 82°38′41.543′′ E, 36°16′6.6′′ N with a migration distance of 24.248 km, and then southeastward to 82°40′19.232′′ E, 36°15′12.28′′ N with a migration distance of 2.962 km. Under the RCP6.0 scenario, it first migrated northwestward to 82°36′42.61′′ E, 36°16′49.634′′ N with a migration distance of 27.12 km, and then turned back southeastward to 82°45′18.241′′ E, 36°11′58.258′′ N with a return distance of 15.721 km. Under the RCP8.5 scenario, the centroid migrated northwestward to 82°37′28.549′′ E, 36°12′50.422′′ N, and then turned back 12 km to 82°45′22.936′′ E, 36°11′32.021′′ N. With the increase in greenhouse gas concentrations, the general direction of centroid change from the present to the future is first moving northeastward and then northwestward, all showing a movement from lower to higher elevation.

### 3.6 Land Use and Habitat Quality Assessment

**FIGURE 15:**
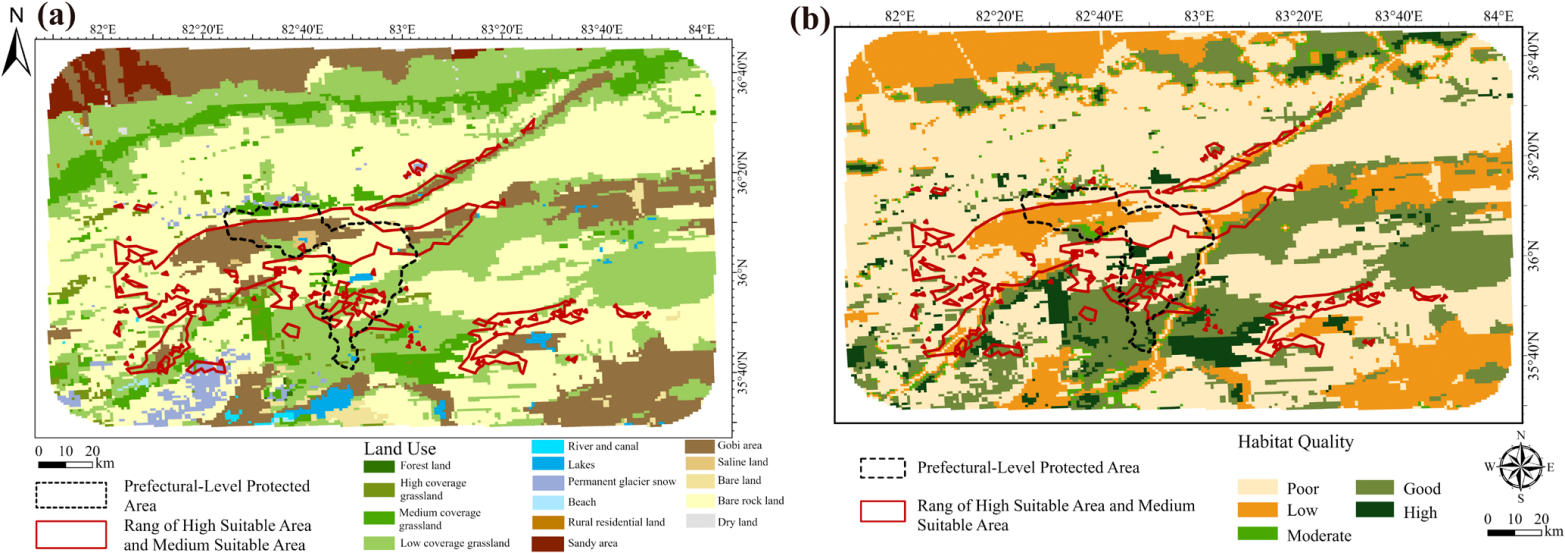
Regional environment. (a) Land use type. (b) Habitat quality.

The land use in the study area is predominantly characterized by medium‐ to low‐coverage grasslands, gobi area, and bare rock land, reflecting a generally fragile ecological environment (Figure 15a). To systematically evaluate the habitat quality within the suitable distribution area of *K. ceratoides* and to identify potential spatial gaps in the conservation of the Tibetan antelope ( *Pantholops hodgsonii* ), the habitat quality index was classified into five grades using the natural breaks method: Poor (≤ 0.145), Low (0.145 < *Q* ≤ 0.306), Moderate (0.306 < *Q* ≤ 0.545), Good (0.545 < *Q* ≤ 0.757), and High (> 0.757) (Figure 15b). A spatial overlay analysis was performed by integrating the moderate‐to‐high suitability zones for *K. ceratoides* and the boundaries of the prefecture‐level reserve designated for Tibetan antelope breeding in the western Kunlun Mountains. The results indicated an overall low habitat quality across the study area, with a mean habitat quality index of only 0.277. High quality habitats were primarily associated with land cover types including grasslands (high, medium, and low coverage), lakes, permanent glacier snow, beach, and forest land. In contrast, both the moderate‐to‐high suitability zones for *K. ceratoides* and the existing reserve are predominantly located in areas with relatively poorer habitat conditions, such as gobi, bare rock land, and low‐coverage grasslands, where the mean habitat quality indices were 0.225 and 0.313, respectively. Notably, the region immediately south of the existing reserve, which is dominated by medium‐ to low coverage grasslands and lakes, exhibits comparatively better habitat quality and is also suitable for the growth of *K. ceratoides* . However, this area currently lies outside the formal reserve boundaries. Considering the foraging habits and dietary preferences of the Tibetan antelope, future conservation planning should prioritize enhanced attention and research focused on this southern area. Its potential inclusion in an expanded conservation network is recommended to address the current spatial inadequacies in protection coverage.

## 4 Discussion

### 4.1 Test of Driving Forces for Suitability of *K. ceratoides*

In this study, we used the biomod2 model to predict the potential distribution areas of *K. ceratoides* during the MH, current, and future climate scenarios, and combined the XGBoost‐SHAP model for cross‐validation of driving forces. Under modern climate conditions, the model exhibited high accuracy, with AUC and TSS values both greater than 0.9, and Kappa value greater than 0.6. The XGBoost‐SHAP model had low RMSE and MAE values, with *R* ^2^ tending to 1 (Figure 6), indicating high reliability of the model predictions. Climatic factors (precipitation, temperature) are the core drivers shaping the distribution of *K. ceratoides* . Among these, the mean diurnal range (bio12) exhibits the highest contribution rate (55.714%), while environmental variables including annual precipitation (bio12), topsoil clay fraction (t_clay), and annual mean temperature (bio1) synergistically regulate its distribution pattern (Figures 5 and 7). Specifically, bio2 serves as a key indicator of plant adaptive capacity to environmental fluctuations: increased diurnal temperature ranges enhance the cold tolerance of alpine plants, define their altitudinal distribution by modulating soil water uptake, and influence plant photosynthetic rates, a mechanism validated in the cushion plant *Azorella compacta* (Kleier and Rundel 2009). Precipitation metrics (bio12, bio17) directly govern water availability, *K. ceratoides* seedlings tolerate mild drought by adjusting fatty acid saturation, but intensified drought disrupts antioxidant enzyme system balance, triggering lipid peroxidation, cell membrane damage, and even cell death; reduced precipitation also elevates stem mortality and accelerates leaf wilting (Ke et al. 2023). Among edaphic factors, t_clay contributes relatively significantly, Higher clay content enhances aggregate stability and noncapillary porosity, thereby promoting root growth (Wang, Su, Zhou, et al. 2023). Alpine vegetation tends to have longer root systems, and higher soil infiltration rates are conducive to plant growth. Analysis of single‐feature dependence plots (Figure 8) reveals complex interactive effects, When bio2 values are high, a decrease in bio12 enhances the positive effect of bio2 on habitat suitability, while the combination of high bio2 and low bio12 leads to arid environments. Similarly, SHAP values are lower in regions with high bio12 and concentrated low bio2, improved environmental conditions intensify interspecific niche competition, yet SHAP values rebound when bio12 increases and bio2 decreases. An increase in t_clay combined with a decrease in bio12 restricts soil water infiltration and increases runoff loss, under intensified drought, plants' ability to extract water from clay soils is further impaired (Gao 2023). When bio1 is within the optimal range, reduced bio12 conversely promotes the distribution of *K. ceratoides* , which is consistent with the findings from coverage surveys of cushion *K. ceratoides* on the Qinghai‐Tibet Plateau (Zhao et al. 2017).

### 4.2 Pattern of Suitable Habitats of *K. ceratoides* Under Different Climate Scenarios

By analyzing the potential distribution of *K. ceratoides* under the MH, current, and future climate scenarios (RCP2.6, RCP4.5, RCP6.0, RCP8.5), we found that its overall range shows an expanding trend but with distinct expansion characteristics: low suitable areas mainly spread outward to surrounding regions, medium suitable areas exhibit limited growth and fragmentation, and high suitable areas almost disappear (Figure 11). Since the Early Holocene, alpine desert plants in western Qinghai‐Tibet Plateau have expanded, alpine grassland area has shrunk, and the climate has tended to aridity (Chen et al. 2020), Zhao et al. (2007) conducted pollen sampling in northern Qinghai‐Tibet Plateau, and results from the sampling site near our study area at the west side of Yanghu Lake (approximate coordinates: 84°22′41.567376′′ E, 35°19′11.865396′′ N) indicated that Chenopodiaceae pollen, which is an indicator of aridity, dominated the region throughout the Holocene., with the paleoenvironment being generally desert and vegetation type being alpine desert, leading to the conclusion that the current west‐to‐east vegetation distribution pattern in northern Qinghai‐Tibet Plateau is consistent with that since the Holocene, thus suggesting *K. ceratoides* may have had a wide distribution during this period. Against the background of climate warming, the overall suitable area of *K. ceratoides* expands under all future climate scenarios (Figures 12 and 13), while our results align with Jandova et al. (2025) finding that climate warming stimulates the growth and reproduction of cushion plants, the key difference is that our study's expansion is primarily concentrated in low suitable areas, with medium suitable areas showing fragmentation and reduced connectivity, high suitable areas nearly vanishing, and climate warming significantly decreasing seed yield of cushion plants which negatively impacting their reproductive success and population survival rate, which is consistent with Cheng's (2022) study on *K. ceratoides* in the Qinghai‐Tibet Plateau regarding changes in suitability patterns. Furthermore, under future climates, the suitable areas of other alpine vegetation in this region, including *Asterothamnus centraliasiaticus*, *Carex moorcroftii*, *Littledalea przevalskyi*, *Littledalea racemosa*, all exhibit expanding trends, mainly in low and medium suitable areas (Li et al. 2024; Liu et al. 2018, 2020), this is highly likely to cause high niche overlap with *K. ceratoides* , intensifying resource competition, and if niche overlap continues to increase, *K. ceratoides* may face extinction risk (Pastore et al. 2021). Wang (2023) employed the MaxEnt model to project changes in the suitable habitat distribution of the Tibetan antelope on the Qinghai‐Tibet Plateau under future climate scenarios. The region currently contains areas of medium to high suitability. Should these areas experience unfavorable shifts in the future, potentially occurring alongside a decline in the medium‐high suitability zones for the cushion plant *K. ceratoides* the breeding populations of Tibetan antelope in this region are likely to face increased threats.

### 4.3 Centroid Migration

This study analyzed the centroid migration characteristics of *K. ceratoides* under different climate scenarios (Figure 14), and the results revealed obvious spatiotemporal heterogeneity in its migration trends: With increasing climate warming, the overall migration direction is from southeast to northwest, and retraction towards the southeast occurs in different periods under future climate scenarios. From the MH to the present, this vegetation shows a trend of migration towards lower elevations during this period, as the vegetation type in the western Qinghai‐Tibet Plateau gradually shifted from grassland to desert. (Huang et al. 1996), and the arid climate restricted the growth of vegetation favoring moisture, prompting *K. ceratoides* with its drought tolerance to spread southward. Under the future climate scenarios of RCP4.5, RCP6.0, and RCP8.5, the centroid basically shows an upward migration trend during 2041–2060. Existing studies (Bentley et al. 2018; Chen et al. 2015) have shown that alpine plants usually adapt to climate warming by migrating to higher elevations or latitudes; however, with intensified warming, extreme precipitation events may trigger floods and landslides on sparsely vegetated slopes, significantly reducing vegetation coverage in alpine and subalpine areas and causing species composition to shift to more humid habitats. The response of plants to climate change is not a simple single upward distribution shift, but a multidimensional, species specific spatial dynamic process (Dolezal et al. 2016). During 2061–2080, the migration of *K. ceratoides* toward lower elevations in the southeast supports this viewpoint: high altitude environments are becoming increasingly unsuitable for vegetation because of frequent extreme climate events, driving species toward more favorable lowlands. Notably, under the low‐concentration scenario of RCP2.6, its centroid migrates eastward, suggesting a preference for areas with more suitable temperature and precipitation conditions.

### 4.4 Environmental Quality

Through comprehensive analysis of the suitable areas (medium and high suitability) of *K. ceratoides* under current climate conditions, the Prefecture‐level Protected Area for Tibetan Antelope Breeding Grounds in the Western Kunlun Mountains, habitat quality, and land use types, the results show that the overall ecological environment of the region is relatively harsh, being dominated by land use types including medium‐low coverage grassland, Gobi land, and bare rock land, and has a mean habitat quality index of only 0.277 (Figure 15). As the frontline of biodiversity conservation and restoration, the protected area requires targeted conservation measures (Hua et al. 2022), as the main summer breeding ground for the western population of Tibetan antelopes ( *Pantholops hodgsonii* ), it has a mean habitat quality index of 0.313, with land use types dominated by Gobi land, bare rock land, and grassland. Notably, the southern part of the protected area has better habitat quality, mostly covered by grassland and relatively suitable for *K. ceratoides* growth, but most of this area is not included in the protected scope. According to Leslie and Schaller's (2008) research on the diet and living environment of Tibetan antelopes, which inhabit alpine and desert steppe regions of the Qinghai‐Tibet Plateau with flat to undulating terrain and feed on herbs, sedges, forbs, and specific parts of dwarf woody vegetation, this unprotected southern area may become a potential key breeding area for Tibetan antelopes and deserves focused attention in the future. Building upon the existing foundation, we will further integrate the use of satellite remote sensing, drone platforms, infrared monitoring, and other technological means to incorporate the Black Stone North Lake area, a region where Tibetan antelope breeding is relatively concentrated, into a long term fixed position monitoring system. We will systematically advance the planning and construction of ecological corridors for Tibetan antelope, thereby effectively reducing the impact of human activities on their habitat and reproduction. In regions with poor habitat quality and low vegetation coverage, such as Gobi land and bare rock land, where the mean habitat quality index is only 0.225, *K. ceratoides* exhibits high adaptability. Gobi deserts mainly host drought tolerant or extremely drought tolerant shrubs and subshrubs, and as one of the harshest natural environments on Earth, plants here have evolved to adapt to extreme conditions (Ding et al. 2022), yet this region, located on the Qinghai‐Tibet Plateau with high altitude and low precipitation, exposes plants to both cold and drought stress, leading to sparse vegetation. The Qinghai‐Tibet Plateau is a hotspot for cushion plant distribution; *K. ceratoides* has traits such as dense branching, compact canopy, spherical morphology, and extensive root systems, which are adaptations to environmental stresses like strong winds, freezing frost damage, and drought (Boucher et al. 2016). Additionally, cushion plants modify the microenvironment: soil temperature under the cushion layer is slightly higher than that of bare ground, water resources within the cushion are more abundant, they mitigate wind erosion, provide favorable microclimates for surrounding alpine plants, and their dead residues supply substrate to the soil and promote nutrient cycling. As key pioneer species and ecological engineers in the alpine ecosystems of the Qinghai‐Tibet Plateau, cushion plants play a crucial role in maintaining the diversity and stability of alpine systems (Arroyo et al. 2003; Wang et al. 2021).

## 5 Conclusion

This study used biomod2 to construct an ensemble model for predicting the potential distribution patterns of *K. ceratoides* under different climate scenarios. The results showed that during the Mid‐Holocene, the species had an extensive suitable habitat area with high overall habitat suitability; currently, its suitable habitats are concentrated in the western and central parts of the study area. Further cross‐validation using XGBoost‐SHAP values revealed that temperature and precipitation are the core environmental factors affecting the distribution of *K. ceratoides* , with mean diurnal range (bio2) exerting the most significant impact. Under future climate scenarios (RCP2.6, RCP4.5, RCP6.0, RCP8.5), although the total suitable habitat area of the species will expand, it will be dominated by low suitable habitats, medium suitable habitats will increase slightly but show fragmented distribution, while high suitable habitats will almost disappear. The migration direction exhibits obvious spatiotemporal heterogeneity: from the MH to the present, it migrated to low elevations; under climate warming, it will first move to uplands in the northwest in the near future, then retreat to lowlands in the southeast over time, facing a high risk of resource competition. In addition, as the main food source for Tibetan antelopes ( *Pantholops hodgsonii* ) in the Western Kunlun Mountains (Yutian and Minfeng County sections), *K. ceratoides* has strong adaptability to harsh environments and can promote the growth and reproduction of surrounding species by regulating microenvironments (e.g., improving soil temperature and moisture conditions), serving as a keystone species in the regional ecosystem. The study also identified conservation gaps for Tibetan antelopes: the southern area near existing protected zones has good habitat quality and is relatively suitable for *K. ceratoides* distribution but has not been included in the protection scope. Based on these findings, future efforts should focus on strengthening monitoring and research on changes in *K. ceratoides* distribution pattern, reducing anthropogenic disturbances, improving planning for vacant areas in protected zones, and enhancing public awareness and educational levels regarding the conservation of *K. ceratoides* and Tibetan antelopes.

## Author Contributions

**Kailing Huang:** conceptualization (equal), data curation (equal), methodology (lead), writing – original draft (lead), writing – review and editing (equal). **Fengbing Lai:** conceptualization (lead), funding acquisition (lead), investigation (equal), resources (lead), visualization (lead), writing – review and editing (equal). **Mengyu Chen:** data curation (lead), investigation (equal), software (lead), supervision (equal), visualization (equal). **Ying Song:** formal analysis (lead), software (equal), visualization (supporting). **Shujiang Chen:** conceptualization (equal), investigation (lead), supervision (lead). **Zubaydah Wubuaysan:** formal analysis (supporting), resources (supporting). **Xiaopeng Zhuang:** data curation (supporting), software (supporting).

## Funding

This research was funded by the National Natural Science Foundation of China (42061003).

## Conflicts of Interest

The authors declare no conflicts of interest.

## Supporting information


**Appendix S1:** ece372900‐sup‐0001‐TableS1‐S4.docx.

**Tables S1–S4:** ece372900‐sup‐0002‐AppendixS1.zip.

## Data Availability

The R code used for all analyses in this study is publicly available on GitHub at https://github.com/huangkailing839‐lgtm/paper.git.
