# Supplementary material for: The Response of Krascheninnikovia ceratoides (L.) Gueldenst. to Environmental Changes Since the Mid‐Holocene in the Tibetan Antelope Breeding Ground of the Western Kunlun Mountains
Source: Ecol Evol. 2026 Jan 16;16(1):e72900. doi: 10.1002/ece3.72900 (PMC12811628; doi:10.1002/ece3.72900)
Supplement: Supplementary file 1 — Appendix S1: ece372900‐sup‐0001‐TableS1‐S4.docx. [file ECE3-16-e72900-s001.docx]

**Appendix**

**TableS1**

Distribution Points Situation

| Longitudes/E | Latitudes/N | elevation/m | source |
| --- | --- | --- | --- |
| 82° 56' 44.79" | 35° 48' 54.16" | 5104 | Field Survey |
| 82° 21' 49.17" | 35° 56' 19.04" | 4528 |  |
| 83° 0' 37.45" | 35° 56' 58.08" | 5114 |  |
| 82° 22' 45.22" | 35° 57' 1.80" | 4644 |  |
| 82° 23' 35.44" | 35° 57' 38.90" | 4813 |  |
| 82° 24' 25.65" | 35° 58' 9.75" | 4890 |  |
| 82° 29' 14.96" | 36° 3' 52.10" | 4567 |  |
| 82° 32' 10.02" | 36° 5' 40.95" | 4568 |  |
| 83° 2' 46.82" | 36° 6' 14.55" | 4903 |  |
| 82° 54' 42.18" | 36° 13' 3.63" | 4984 |  |
| 83° 0' 56.79" | 36° 15' 3.66" | 4795 |  |
| 83° 19' 19.60" | 36° 25' 3.48" | 4138 |  |
| 82° 30' 34.55" | 36° 4' 34.19" | 4564 |  |
| 82° 53' 32.69" | 82° 53' 32.69" | 5065 |  |
| 83° 2' 21.73" | 36° 15' 24.90" | 4717 |  |
| 83° 26' 14.14" | 36° 29' 31.41" | 3860 |  |
| 83° 3' 57.16" | 36° 21' 18.40" | 5646 | Paper |
| 83° 47' 27.60" | 35° 51' 50.40" | 5086 | GBIF |

**TableS2**

Threats Table

| MAX_DIST | WEIGHT | THREAT | DECAY |
| --- | --- | --- | --- |
| 5 | 0.6 | Bare land | Linear |
| 3 | 0.7 | Dry land | Linear |
| 3 | 1 | Grazing | Linear |
| 3 | 0.8 | Road | Exponential |
| 8 | 1 | Rural settlements | Exponential |
| 5 | 0.6 | Saline land | Linear |

**TableS3**

Sensitivity Table

| lulc | NAME | HABITAT | Bare land | Dry land | Grazing | Road | Rural settlements |
| --- | --- | --- | --- | --- | --- | --- | --- |
| 12 | Dry land | 0.4 | 0.5 | 0.25 | 0.2 | 0.4 | 0.4 |
| 21 | Forest land | 1 | 0.9 | 0.8 | 0.6 | 0.8 | 0.7 |
| 31 | High coverage grassland | 0.9 | 0.2 | 0.7 | 0.8 | 0.8 | 0.7 |
| 32 | Medium coverage grassland | 0.8 | 0.3 | 0.7 | 0.7 | 0.8 | 0.7 |
| 33 | Low coverage grassland | 0.7 | 0.3 | 0.7 | 0.7 | 0.8 | 0.7 |
| 41 | River and canal | 1 | 0.5 | 0.6 | 0.7 | 0.7 | 0.8 |
| 42 | Lakes | 1 | 0.6 | 0.7 | 0.75 | 0.8 | 0.8 |
| 44 | Permanent glacier snow | 0.6 | 0.6 | 0.3 | 0.2 | 0.6 | 0.6 |
| 46 | Beach | 0.6 | 0.4 | 0.6 | 0.6 | 0.7 | 0.8 |
| 52 | Rural residential land | 0 | 0 | 0.1 | 0.1 | 0.1 | 0 |
| 61 | Sandy area | 0.2 | 0 | 0 | 0.2 | 0.2 | 0 |
| 62 | Gobi area | 0.2 | 0.2 | 0.2 | 0.3 | 0.4 | 0.4 |
| 63 | Saline land | 0.2 | 0 | 0.1 | 0.2 | 0.5 | 0.5 |
| 65 | Bare land | 0.1 | 0 | 0 | 0.2 | 0.4 | 0 |
| 66 | Bare rock land | 0.1 | 0.1 | 0.1 | 0.1 | 0.3 | 0.2 |

**TableS4**

Centroid positions and elevation of suitable habitats of *K. ceratoides* under different climate scenarios

| climate scenario | Longitudes/E | Latitudes/N | Elevation/m |
| --- | --- | --- | --- |
| MH | 82°40'56.348" | 36°16'29.24" | 5174 |
| Current | 82°48'11.106" | 36°5'31.07" | 4684 |
| RCP2.6-2050S | 82°56'43.267" | 36°12'1.868" | 5086 |
| RCP2.6-2070S | 82°58'12.151" | 36°12'25.816" | 5061 |
| RCP4.5-2050S | 82°38'41.543" | 36°16'6.6" | 5429 |
| RCP4.5-2070S | 82°40'19.232" | 36°15'12.28" | 5966 |
| RCP6.0-2050S | 82°36'42.61" | 36°16'49.634" | 4894 |
| RCP6.0-2070S | 82°45'18.241" | 36°11'58.258" | 4834 |
| RCP8.5-2050S | 82°37'28.549" | 36°12'50.422" | 5408 |
| RCP8.5-2070S | 82°45'22.936" | 36°11'32.021" | 4764 |
